# Assessment of quality and pre-clinical efficacy of a newly developed polyvalent antivenom against the medically important snakes of Sri Lanka

**DOI:** 10.1038/s41598-021-97501-2

**Published:** 2021-09-14

**Authors:** Aparup Patra, Bhargab Kalita, Milind V. Khadilkar, Nitin C. Salvi, Pravin V. Shelke, Ashis K. Mukherjee

**Affiliations:** 1 Microbial Biotechnology and Protein Research Laboratory, Department of Molecular Biology and Biotechnology, School of Science, Tezpur University, Tezpur, Assam 784028 India; 2 Premium Serums and Vaccines Pvt. Ltd, Narayangaon, Pune, Maharashtra 410504 India; 3 Institute of Advanced Study in Science and Technology, Vigyan Path, Garchuk, Paschim Boragaon, Guwahati, Assam 781035 India

**Keywords:** Biochemistry, Biological techniques, Health care, Medical research

## Abstract

Snake envenomation is a severe problem in Sri Lanka (SL) and Indian polyvalent antivenom (PAV) is mostly used for treating snakebite albeit due to geographical variation in venom composition, Indian PAV shows poor efficacy in neutralizing the lethality and toxicity of venom from the same species of snakes in SL. Therefore, the quality and in vivo venom neutralization potency of a country-specific PAV produced against the venom of the five most medically important snakes of SL (*Daboia russelii, Echis carinatus, Hypnale hypnale, Naja naja, Bungarus caeruleus*) was assessed. LC-MS/MS analysis of two batches of PAV showed the presence of 88.7–97.2% IgG and traces of other plasma proteins. The tested PAVs contained minor amounts of undigested IgG and F(ab′)_2_ aggregates, showed complement activation, were devoid of IgE, endotoxin, and content of preservative was below the threshold level. Immunological cross-reactivity and in vitro neutralization of enzymatic activities, pharmacological properties demonstrated superior efficacy of SL PAV compared to Indian PAV against SL snake venoms. The in vivo neutralization study showed that the tested PAVs are potent to neutralize the lethality and venom-induced toxicity of SL snake venoms. Therefore, our study suggests that introduction of SL-specific PAV will improve snakebite management in SL.

## Introduction

Snakebite is a severe problem in the Indian subcontinent and has been declared as a neglected tropical disease by the World Health Organization (WHO). Sri Lanka (SL), a small island of South Asia located in the Indian Ocean, reports approximately 30,000 snake envenomations that result in 450 deaths per year^1,2^. SL is the home for 96 terrestrial and 15 aquatic species of snakes^3^, with the hump-nosed viper (*Hypnale hypnale*)*,* Russell’s viper (*Daboia russelii*)*,* the Indian common krait (*Bungarus caeruleus*)*,* the Indian cobra *(Naja naja)*, and the saw-scaled viper (*Echis carinatus*) are regarded as the most dangerous species of venomous snakes since they account for most of the snakebite fatalities in SL^4^.

Snakebite is a medical emergency that requires immediate medical treatment; otherwise, it may result in death and/or permanent morbidity. The only effective treatment against snakebite is intravenous administration of polyvalent antivenom (PAV). In SL, the most widely used antivenom is the Indian PAV raised against the ‘big four’ venomous snakes of India (*D. russelii*, *N. naja*, *E. carinatus,* and *B. caeruleus*)^5^. Currently, important concerns are being raised about using Indian PAV for the treatment of snakebites in SL. The primary objection is that due to geographical variations, the composition of Indian snake venoms used for immunizing and raising equine antibodies are different from the venom compositions of the same species of snakes in SL^6–10^. Consequently, Indian PAV is found to be less efficient in neutralizing the lethality and toxicity of venoms of SL snakes and additional doses of PAV are necessary for treating snakebite patients in SL. This increases the risk of adverse serum reactions in the antivenom-treated patients^11,12^ and unnecessarily increases the cost of treatment. The Indian PAVs are also limited by its absence of antibody against *H. hypnale* venom (HHV), which is responsible for the most envenomation in SL^4^. Neutralizing HHV is therefore exclusively dependent on the antibodies raised against the venom toxins of the same Viperidae family of snakes (*D. russelii* and *E. carinatus*), and consequently, the PAV that is in current use is less efficient for treating *H. hypnale* envenomation in SL^12^. Further, the supply of PAV from India is not sufficient to fulfill the annual requirements for antivenom in SL, which is causing a shortage of antivenom in this country^4^. These issues can be addressed by developing SL-specific PAV raised against snake venoms of SL origin, which is urgently needed for effective snakebite treatment in this country.

Recently, the pre-clinical efficacy of a newly developed SL venom-specific antivenom was reported^13^; however, a similar initiative is still desired to fulfill the demand for PAV to treat the large number of snakebites in SL. In this study, we characterized a newly developed country-specific PAV designated as SL PAV against *H. hypnale* venom (HHV)*, D. russelii* venom (DRV)*, N. naja* venom (NNV)*, E. carinatus* venom (ECV), and *B. caeruleus* venom (BCV) sourced from SL. Notably, the World Health Organization (WHO) Guidelines for the Production, Control and Regulation of Snake Antivenom Immunoglobulins published in 2016, recommended that standard in vitro laboratory tests be conducted to ascertain the purity, quality, and safety of the antivenom as the essential quality control before the pre-clinical assessment of PAV^14,15^. Therefore, in addition to assessing the in vivo neutralization potency of SL snake venoms-induced lethality and toxicity by SL PAV, we also determined the physicochemical properties, purity of active substance, load of preservatives and neutralization of some enzymatic activity and pharmacological properties of SL snake venoms by this newly developed SL PAV by in vitro laboratory assessments. Our study has shown that some pharmacological properties of SL snake venoms and their enzymatic activity were neutralized by the newly developed SL PAV to a greater extent when compared to Indian PAVs that are being used for snakebite treatment in SL. The assessment indicated the enhancement of efficacy and potency of the former PAV.

## Results and discussion

### Preliminary physicochemical analysis of SL PAV

Examining the physicochemical properties of a drug is a preliminary but an important aspect for assessing its quality. Both vials of lyophilized PAV appeared as homogenous white colored powder, with fragment cake-like structure (Supplementary Fig. S1); however, batch 1 (B1) showed more shrinkage than the batch 2 (B2). These cake-like structure may be formed due to mechanical damage and may have impact on reconstitution time and turbidity^16^ but the cake-like structure of the lyophilized powder does not affect their biological activity (venom neutralization potency)^17^. The SL PAV was found to be completely dissolved within 5–6 min in 10 mL of sterile deionized water and no insoluble material was observed. This result suggests good water solubility of lyophilized powder. The turbidity measures the cloudiness of a solution; therefore, low turbidity of a solution indicates that solute (PAV in this case) is well mixed with the solvent (sterile deionized water). Consequently, WHO has suggested for quantitative measurement of the turbidity of antivenoms solution by using a turbidimeter; albeit, no standard value of turbidity is fixed^14^. The turbidity of the SL PAV B1 and B2 was found to be 15.3 ± 0.6 and 11.1 ± 0.8 nephelometric turbidity units (NTUs), respectively indicating the good solubility of lyophilized powder in water. The pH of the reconstituted PAV should be around 7.0 ± 0.5^14^. The aqueous solutions of the two batches of SL PAVs had pH values between 6.95 ± 0.2 and 6.89 ± 0.3, which is quite close to the standard set by the WHO.

In developing countries, snakebite is regarded as an occupational health hazard for rural people and a socio-economic problem^18^, and therefore, PAV should be available at rural health centers. The lyophilized PAV is more suitable than liquid antivenom for long-term storage (2–3 years) without requiring refrigeration at rural hospitals. The moisture content of the lyophilized product reduces the shelf-life either by denaturing the product or by aggregating F(ab′)_2_ molecules that causes a significant reduction in its venom neutralization potency^14^. For the two batches of the tested SL PAVs, the moisture content was determined to be in the safe range (< 3%)^14^.

### Tandem mass spectrometry and size exclusion chromatography analyses to determine the purity of the active substances of PAV preparations

The antivenoms are manufactured as undigested immunoglobulin G (IgG) molecules^13^ or fragmented F(ab′)_2_ molecules (pepsin digested IgG to remove its Fc region)^19^. The Indian PAVs and the newly developed SL PAV contain F(ab′)_2_ and therefore theoretically believed to show less serum reactions in treated patients, however, detailed clinical studies are warranted to confirm the advantages of pepsin digested IgG^14,20^. The neutralization potency and efficacy of IgG and F(ab′)_2_ are not significantly different; however, they have different pharmacokinetic profiles^14,21^. Notably, F(ab′)_2_ has certain advantages over the parent IgG molecule, for example, easier access to extravascular tissue and rapid elimination from the blood^14,21^, higher distribution rate and neutralization potency for low molecular mass venom toxins^14,22^ particularly against the venoms of Elapidae family of snakes comprised of relatively higher proportion of low molecular mass toxins^23–25^.

**Figure 1:**
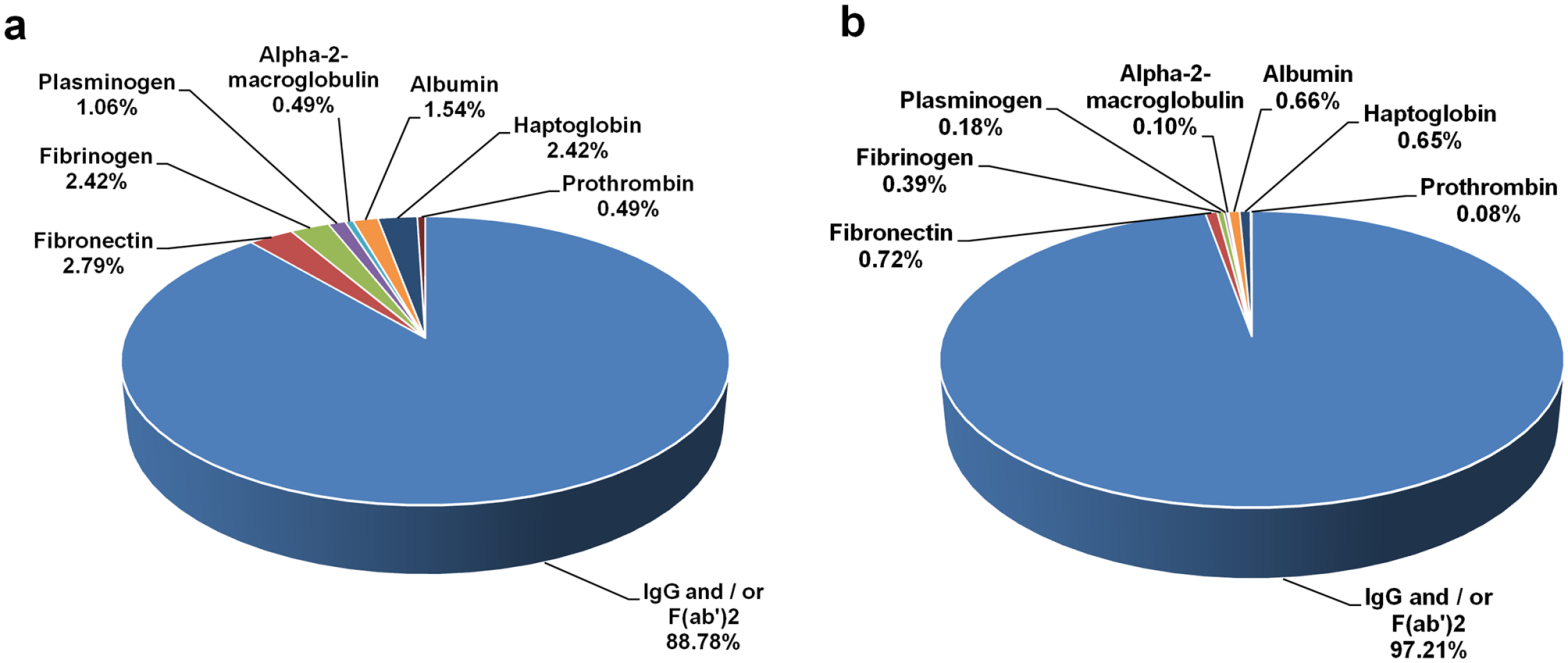
Compositional analysis of two batches of SL PAV determined by LC–MS/MS analysis. (**a**) B1 and (**b**) B2.

One of the key steps in the antivenom manufacturing process is the caprylic acid treatment of horse hyper-immunized serum to precipitate the non-IgG proteins^14^. Because experimental error or insufficient quality control during the preparation might lead to incomplete or partial digestion of IgG proteins or contamination of F(ab′)_2_ with the undesirable non-IgG proteins of serum, the SL PAV was analyzed by LC–MS/MS to determine the presence of any contaminating serum proteins^19^. According to WHO guidelines, total protein concentration of PAV solution should not exceed 10 g/dL^14^. The protein concentration of the aqueous solution of B1 and B2 of SL PAV was found to be 7.3 ± 0.2 and 7.5 ± 0.4 g/dL, respectively indicating they accomplish the WHO guidelines^14^. The LC–MS/MS analysis of the SL PAV batches against *Equus caballus* (taxid 9796) proteins in the UniProt database by using emPAI-based label-free quantification method showed that the percent content of immunoglobulin molecule [IgG and/or F(ab′)_2_] in B1 and B2 of SL PAV was 88.8% and 97.2%, respectively, and the remaining contaminating proteins were fibronectin, fibrinogen, haptoglobin, serum albumin, alpha-2 macroglobulin, albumin, prothrombin, and plasminogen (Fig. 1, Supplementary Table S1). The above mentioned contamination proteins are also found to be present however to a varying extent in other commercial PAVs^19,26–28^ suggesting improved cost-effective method for the isolation of IgG and/or F(ab′)_2_ is required. As per the recommendation of WHO (2016)^14^, the albumin concentration should not be more than 1% of total protein content of antivenom. In the present study, the albumin content of B2 is found within this limit however, this content is slightly higher in B1 (Fig. 1). Notably, ion-exchange chromatography of hyper-immunized serum after caprylic acid precipitation has been suggested to significantly reduce the contaminating proteins in PAV preparations^29^. However, adopting this process for antivenom manufacturing, which is not required according to the WHO guidelines, would increase the production cost of PAV and make it less affordable in the developing nations.

The purity of the active substances [F(ab′)_2_] of both batches of SL PAV was assessed by gel filtration chromatography (GFC) followed by SDS-PAGE analysis of the gel filtration protein peaks. The gel filtration chromatogram of each batch of SL PAV was resolved into a major broad protein peak (GF1) that eluted around fractions 42–68 mL (Supplementary Fig. S2). The percent protein content of GF1 in the two different batches of SL PAV was determined to be in the range from 93.7 to 96.8% (Supplementary Fig. S2). When compared to the standard chromatograms of purified F(ab′)_2_ and horse IgG, the GF1 fraction of SL PAV batches 1 and 2 were found to contain F(ab′)_2_ and/or IgG (Supplementary Fig. S2); however, minor quantities of other contaminating serum proteins with a molecular mass close to that of IgG or F(ab′)_2_ may also be present in this fraction that can only be determined by the LC–MS/MS analysis of the GF fractions.

**Figure 2:**
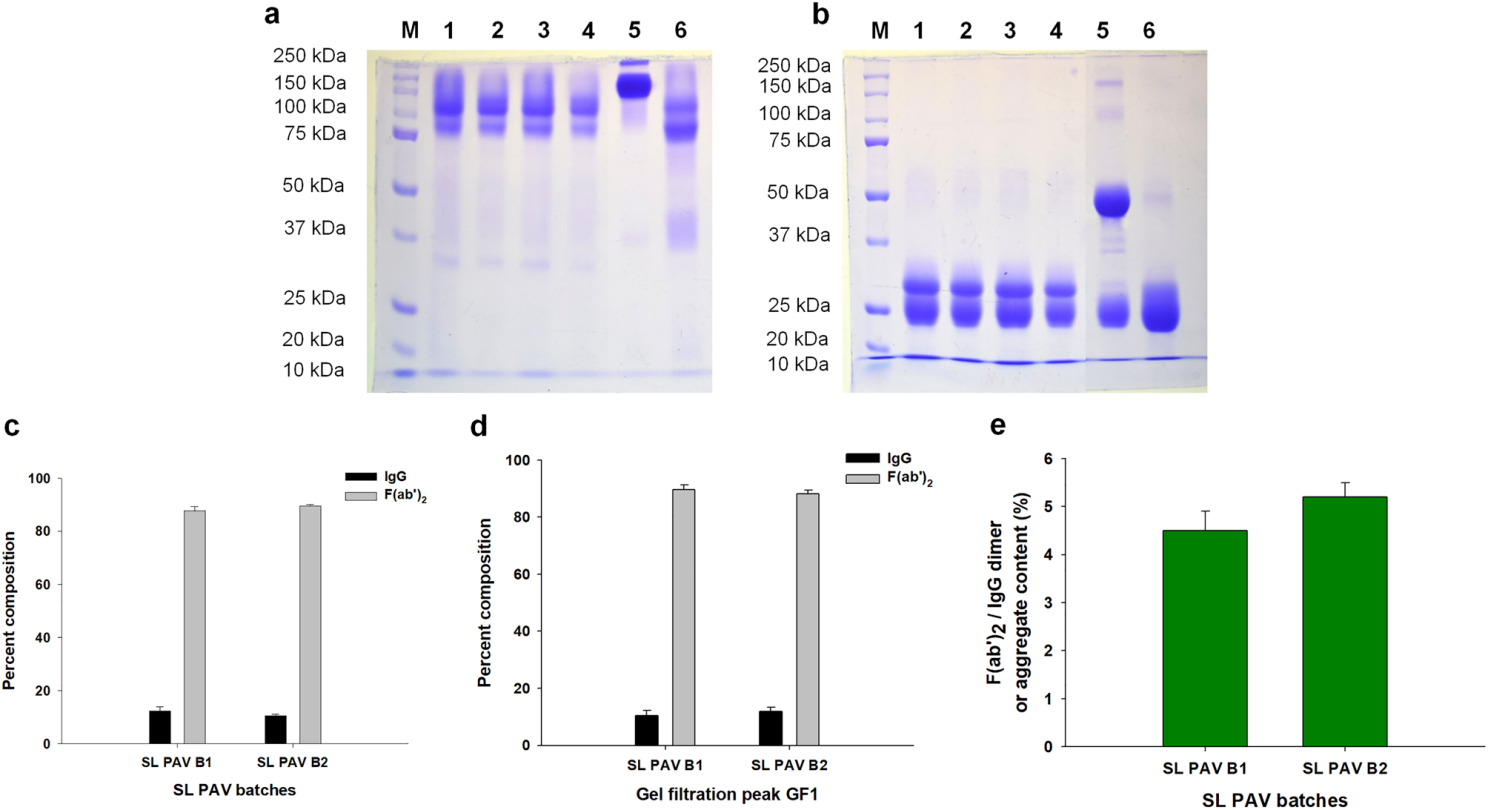
SDS-PAGE analysis of the two batches of SL PAV and their gel filtration fractions under (**a**) non-reduced, and (**b**) reduced conditions. Lanes M, protein molecular markers; lanes 1 and 3 represent PAV of batch B1 and B2, respectively; lanes 2 and 4 represent gel filtration peak GF1 of B1 and B2 of PAV, respectively; lanes 5 and 6 represent purified horse IgG and F(ab′)_2_ fragment, respectively. Determination of percent composition of IgG and F(ab′)_2_ by densitometry analysis; (**c**) two batches of SL PAV and (**d**) their gel filtration fractions (GF1). (**e**) Analysis of F(ab′)_2_ and/or IgG aggregates in the PAVs (B1 and B2) by SDS-PAGE analysis. Values are mean ± SD for triplicate determinations. No significant difference was observed between the two batches of SL PAV (p > 0.05).

The SDS-PAGE analyses of both batches of SL PAV and their GF peak 1 under non-reduced and reduced conditions are shown in Fig. 2a,b. Under the non-reduced conditions, the PAV samples and their GF peaks showed a smeared band at molecular mass ranging from 100 to 170 kDa (Fig. 2a) which is due to undigested IgG and/or aggregates of IgG and/or F(ab′)_2_. In reduced condition, affinity-purified horse F(ab′)_2_ resolved with a single band ~ 25 kDa as both the heavy and light chains are of same molecular mass (25 kDa); however, the tested PAVs showed a major broad band at ∼ 25 kDa along with a light-stained band at ∼ 35 kDa suggesting some of the F(ab′)_2_ molecules were incompletely digested in the PAV preparation (also see below) (Fig. 2b). This findings were also similar to the previously characterized F(ab′)_2_ antivenom from different manufacturers^16,19,26,28,30^. Therefore, the pepsin digestion protocol should be properly followed and special attention should be given to avoid incomplete digestion of IgG molecules.

The densitometry analysis of the SDS-PAGE protein bands of both batches of SL PAV and their respective GF peak (GF1) suggested that they contained minor amounts of undigested IgG (10.0–12.2%) along with F(ab′)_2_ molecules (87.8–89.9%) (Fig. 2a–d). The other minor faint protein bands observed in SDS-PAGE may correspond to the other contaminating serum proteins identified by LC–MS/MS analysis. The SDS-PAGE analysis of the two batches of SL PAVs showed a minor amount of aggregate (4.5 and 5% for B1 and B2, respectively) content (Fig. 2e). This result corroborates the characterization of SABU Indonesian antivenom that showed 7.3% of the F(ab′)_2_ dimer and aggregates in the antivenom preparation^26^; however some of the Indian PAVs showed more aggregate (10–30%) content^28^. The protein aggregates are responsible for early adverse reactions^14^, though customization and refinements in the antivenom production protocol can lead to pure, aggregate-free F(ab′)_2_ antivenom^31^.

### Characterization of the safety profiles of SL PAV

**Figure 3:**
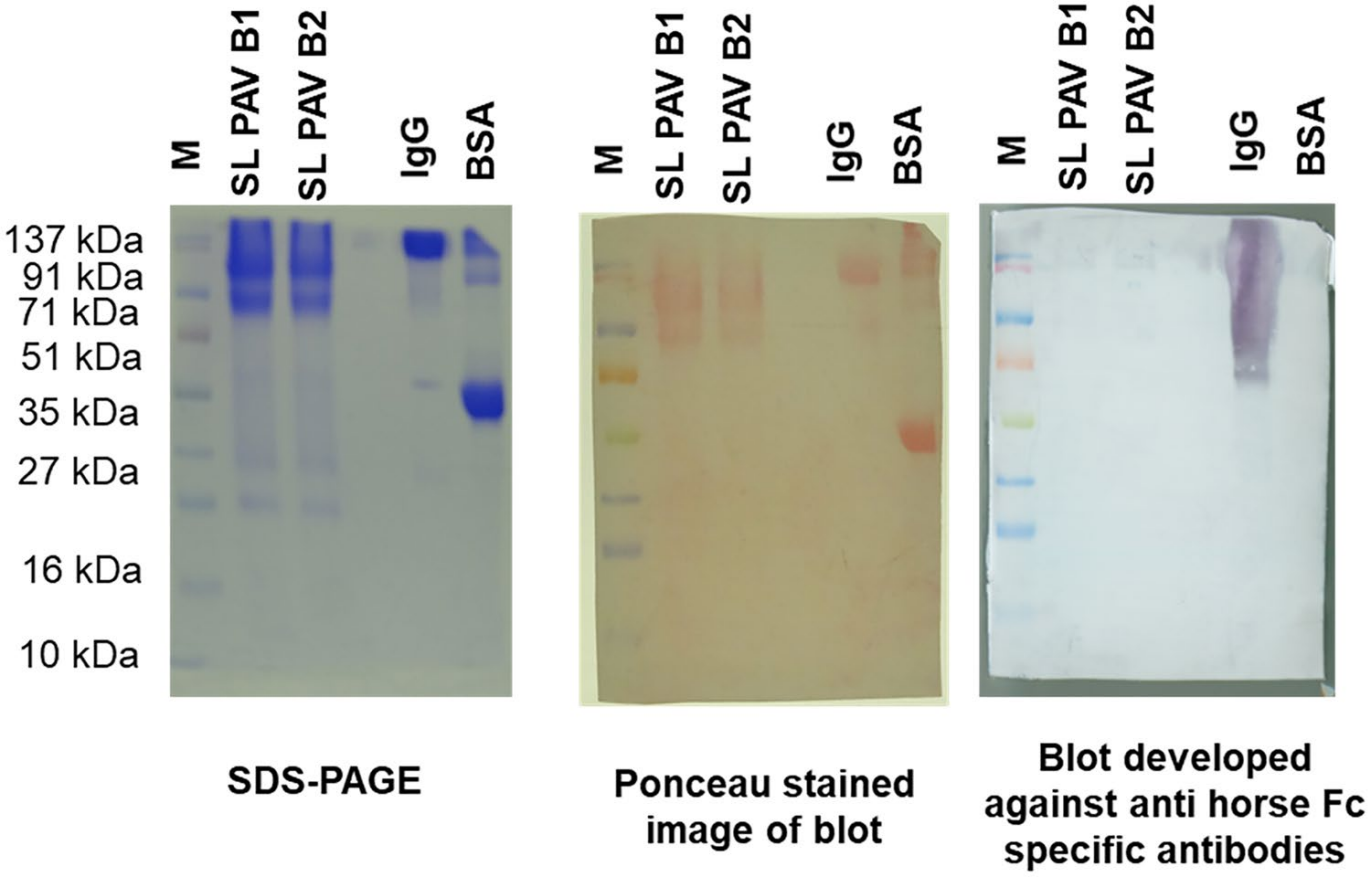
Determination of Fc content of IgG by Western blot analysis. Immunoblot analysis of the SL PAV B1, B2 and purified horse IgG was done by using anti horse IgG HRP conjugated Fc region-specific antibody. M represents protein molecular mass marker and B1 and B2 represent batch1 and batch 2 of PAV, respectively. The primary antibody for both the analyses was raised against Fc region of IgG.

The presence of incomplete or partially digested IgG in SL PAV was determined by enzyme-linked immunosorbent assay (ELISA) and western blot analyses using anti-Fc antibody. The ELISA and immunoblot analysis of the two batches of SL PAV showed 13.9 ± 0.6% and 12.1 ± 0.5% (B1 and B2, respectively) of undigested IgG content (Fig. 3). This result is corroborated by the data from the densitometry analysis of SDS-PAGE bands of crude SL PAV (Fig. 2c). Although the venom neutralization potency is the same for IgG and F(ab′)_2_, the latter product shows less complement activation^14,32^. While partially digested IgG was present in the PAV in this study, further precision is recommended in this step to improve the product quality.

The complement activation property of antivenom is primarily due to the Fc portion of the undigested IgG^14,33^, however, as already discussed removal of Fc portion of IgG by pepsin digestion does not guarantee the elimination of early anaphylactic reactions (EARs) induced by complement activation^34^. Studies have shown that, both IgG and F(ab′)_2_ antivenoms showed higher complement activation when precipitated with ammonium sulphate salt compared to precipitation by caprylic acid^34,35^. Nevertheless, IgG/F(ab′)_2_ aggregates, an excess amount of preservative (cresol), and the hinge portion of the IgG can also contribute to complement activation in PAV-treated patients^33,36,37^. The two batches of SL PAV showed complement activation in vitro with a CH50/mL value (%) of 61.6 ± 3.2, and 64.5 ± 2.9 and AP50/mL value (%) 69.1 ± 4.1 and 65.7 ± 3.9 for SL PAV B1 and B2, respectively (Supplementary Fig. S3). Antivenoms manufactured by Butantan Institute (São Paulo, Brazil) and Clodomiro Picado (San José, Costa Rica) were reported to have variable CH50/mL values (50–95%), which would account for variable degrees of complement activation in vitro^37^. A recent study has shown the CH50/mL and AP50/mL values (%) of Indian PAVs in the range of 45 to 70% and 30 to 60%, respectively^28^. Both the IgG and F(ab′)_2_ molecules can activate the complement pathway, though the former compared to later showed higher complement activation in vitro^14,32^. Noteworthy to mention, the WHO has not fixed any standard safe range for complement activation by antivenoms^15^. The complement activation shown by SL PAV in this study was assessed only by in vitro analysis and this should be confirmed and validated by in vivo pre-clinical studies or clinical case studies from antivenom-treated patients.

IgA and IgE molecules can be co-separated with the IgG molecule during precipitation of IgG from hyper-immune plasma and have no role in neutralizing the venom toxin^14,38^. Although no adverse effect of IgA has been reported, IgE contamination in antivenom preparations can induce a hypersensitivity reaction leading to anaphylactic shock in antivenom-treated patients^39^. The two batches of SL PAV were found to contain IgA (Supplementary Fig. S4a–c) but they were devoid of IgE contamination.

The sterility assessment of SL PAV showed that both batches were free of microbial contamination (Supplementary Fig. S5). During the processing of immunoglobulins of horse plasma, they may become contaminated with bacteria, and as a consequence, PAV can be contaminated with bacterial endotoxin^40^. Depending upon the potency and quality of product, the doses of antivenom ranges from 20 to 120 mL and the maximum acceptable content of endotoxin in an antivenom preparation should be within a range of 2.9 to 17.5 EU/mL (endotoxin unit/mL or EU/mL) and above this level, a high risk exists for inducing pyrogenic reaction in antivenom-treated patients^41^. The endotoxin load in the two batches of SL PAVs was determined to be within the range of 1.5–1.7 EU/mL, suggesting that they are safe (in terms of endotoxin contamination) to administer to snakebite patients (Supplementary Fig. S6a,b).

Cresol is used as preservative for long-term storage of antivenoms; however, the cresol content in antivenom should not exceed 0.35% by weight (or 3.5 g/L)^14^. A high content of cresol in antivenoms has been reported to cause a hypersensitive reaction in patients^33^. RP-HPLC analysis showed that the cresol content in the two batches of SL PAV was within the range of 0.18 to 0.23% (Supplementary Fig. S7a–c), which is significantly below the limit of allowed preservative in an antivenom preparation. Thus, these results advocate for the quality and safety of the newly developed SL PAV.

The number of vials of PAV required to cure a snakebite patient depends on the severity of the envenomation (amount of venom injected into the victim), efficacy of the antivenom, and sometimes repeated doses of PAV are administered over a period of 2–3 days. Therefore, to determine any possible deleterious effects of PAV, mammalian cells were challenged with a high dose (20 µg/mL or 2 mg/dL) of SL PAV and cytotoxicity, if present, was assessed up to 72 h post treatment. None of the tested mammalian cells showed cytotoxicity (Supplementary Fig. S8). Furthermore, both batches of PAV were devoid of direct or indirect hemolytic activity against mammalian erythrocytes (data not shown).

A summary of the physicochemical properties and safety profiles of the two batches of SL PAV is shown in Table 1.

**Table 1:** Summary of the quality and safety assessment of SL PAV batch 1 and batch 2.

Parameters (unit)	Testing method	Results		Regulatory requirement (WHO, 2016)^14^
		SL PAV B1	SL PAV B2	
Appearance	Visual observation of colour, and physical appearance of the powder (in case of freeze-dried preparations)	White in colour and powder like appearance, more cake-like structure is observed	White in colour and powder like appearance, less cake-like structure than B1	Compliance with the description of the marketing dossier
Residual moisture (freeze-dried preparations) (%)	Heat dying method	< 3	< 3	Less than 3%
Solubility (freeze-dried preparations) (min)	Addition of solvent and observation of time to reach solubility and of appearance	~ 5	~ 5	Product should be completely dissolved within 10 min
Turbidity (NTU)	By using a turbidimeter	15.3 ± 0.6	11.1 ± 0.8	Solution should not be cloudy but threshold level is not mentioned in the guidelines
pH of solution	By using a potentiometer (pH meter)	6.95 ± 0.2	6.89 ± 0.3	Specifications of Pharmacopeias and regulatory agencies (generally neutral pH)
Total protein concentration (g/dL)	Lowry method	7.3 ± 0.2	7.5 ± 0.4	Not more than 10 g/dL
Serum albumin content (%)	LC–MS/MS	1.54	0.66	Ideally should be within 1%
Test for pyrogen substances (EU/mL)	Limulus Amebocyte Lysate (LAL) test when validated and approved by national regulatory agency	1.55 ± 0.07	1.8 ± 0.05	Accepted limits by the pharmacopeia in use
Sterility test (microbial cfu)	Filtration through membranes, neutralization (when preservatives are used), and addition to culture media (trypticase soy broth and thioglycolate)	No microbial growth	No microbial growth	Absence of microbial growth
Concentration of preservative (g/L)	RP-HPLC-based method (cresol)	0.4 ± 0.03	0.3 ± 0.02	Phenol: maximum 2.5 g/l
				Cresol: maximum 3.5 g/l
Complement activation (CH50/mL and AP50/mL)	Biochemical assay to determine CH50 and AP50 value for classical and alternative pathway, respectively	CH50/mL (%)–61.6 ± 3.2	CH50/mL (%)–64.5 ± 2.9	Threshold level is not mentioned
		AP50/mL (%)–69.1 ± 4.1	AP50/mL (%)–65.7 ± 3.9	
Fc content of IgG (%)	Immunological assay (ELISA and immunoblotting) by using anti-Fc specific antibody	13.9 ± 0.6	11.8 ± 0.5	Threshold level is not mentioned

Detailed methodologies are described in the text.

### Comparison of immunological cross-reactivity and neutralization of enzyme activity and pharmacological properties of SL snake venoms by Indian and SL PAV

For treating snakebite in SL, Indian PAV is mostly used, though its efficacy and neutralization potency towards SL snake venoms is of immense concern^13,42^. Further, Indian PAV does not contain antibody against HHV, even though the largest number of snake bites in SL are from this species^3,4^. Therefore, it would be essential to include HHV-specific antibodies in a PAV preparation for better protection against snakebite by this pit viper in SL. This long-standing requirement of clinicians can be fulfilled by the newly developed SL PAV. The superiority of the newly developed country-specific SL PAV over Indian PAV was demonstrated by ELISA and Western blot analyses, two important laboratory techniques for quantifying the venom-antivenom cross-reactivity^43–47^. Although in vivo neutralization study of venom-induced lethality and toxicity is the gold standard for pre-clinical assessment of a PAV; however, prior to the in vivo pre-clinical assessment, the efficacy and potency of the antivenom can be assessed by determining the immunological cross-reactivity between venom and antivenom (by ELISA and immuno-blotting techniques). The in vitro neutralization of selected enzymatic activities and pharmacological properties of venom samples can also be evaluated. The results of these tests can provide a clear understanding of the efficacy of antivenom. These assessments are highly recommended as they involve a minimal use of experimental animals and they are ethically acceptable^19,44,46–53^.

**Figure 4:**
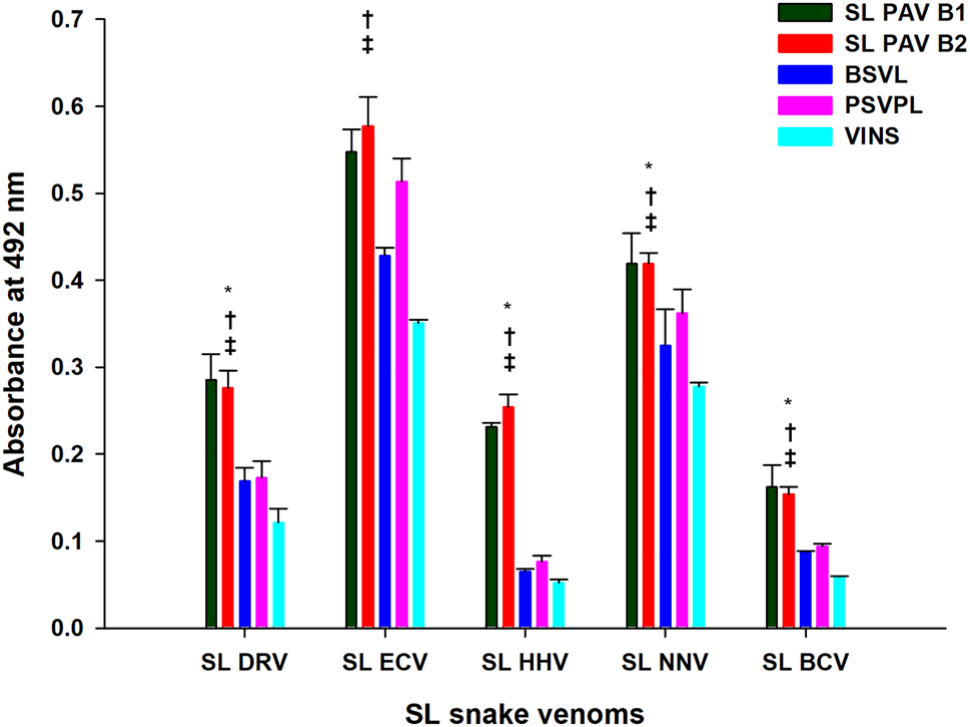
Determination of immunological cross-reactivity between two batches of SL PAV/Indian PAVs and SL snake venoms by ELISA. Values are mean ± SD for triplicate determinations. *Significance of difference between SL PAV B1 and B2 with respect to BSVL; ^†^significance of difference between SL PAV B1 and B2 with respect to PSVPL; ^‡^significant difference between SL PAV B1 and B2 with respect to VINS, (p < 0.05).

The result of the ELISA showed that both batches of SL PAV (compared to Indian PAV) demonstrated significantly higher immuno-recognition of the SL snake venom samples; PAVs demonstrated the highest and lowest immuno-recognition against the venom of *E. carinatus* and *B. caeruleus*, respectively (Fig. 4). Further, the two batches of SL PAV did not show any significant deviation (p > 0.05) in their immuno-recognition of venom samples. In addition, because Indian PAVs do not contain antibodies against HHV, their recognition towards HHV (compared to that of SL PAV) was extremely poor (Fig. 4). Though, a marginal recognition of *H. hypnale* by Indian PAVs suggests that these venom toxins may share sequence similarities with the homologous toxins from Indian *D. russelii* and *E. carinatus* venoms as they all belong to the Viperidae family of snakes^6,44,45,49,54^.

**Figure 5:**
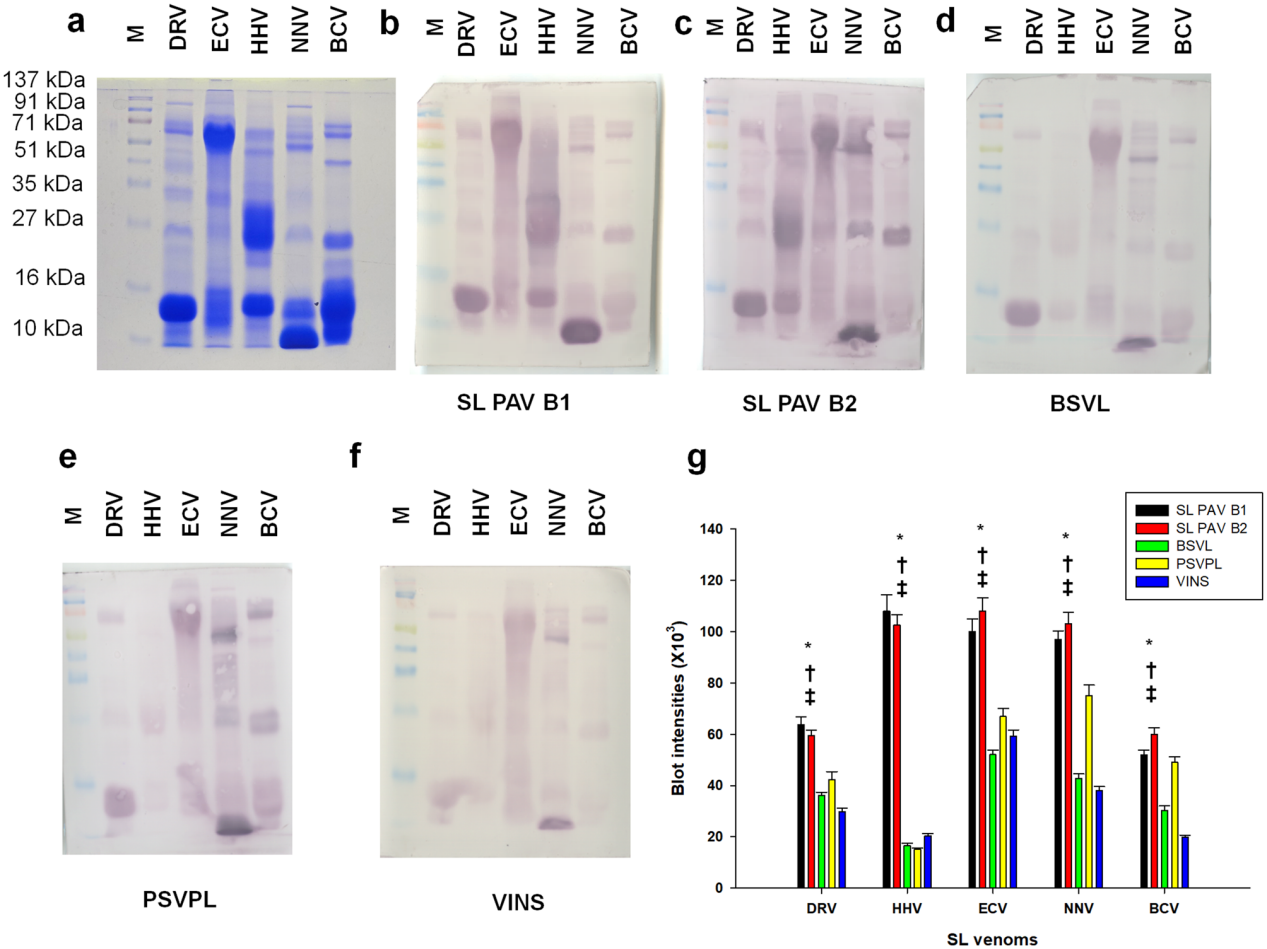
Immunoblot cross-reactivity of SL venoms. (**a**) SDS-PAGE images of SL venoms. (**b**–**f**) Western blot images of SL venoms against two batches (B1 and B2) of SL PAV, and Indian PAVs (BSVL, PSVPL, and VINS). (**g**) Densitometry analyses of immunoblots as shown in (**b**–**f**) Values are mean ± SD of duplicate determinations. *Significant difference between SL PAV B1 and B2 with respect to BSVL; ^†^Significant difference between SL PAV B1 and B2 with respect to PSVPL; ^‡^Significant difference between SL PAV B1 and B2 with respect to VINS (p < 0.05). Same molecular marker (M) was used for all the blots. The Ponceau stained blot images are shown in supplementary Fig. S8. The full length unedited blot images are shown in supplementary Fig. S9.

Several researchers have suggested that immunoblot analysis is an important in vitro laboratory test to determine the potency of PAV^45,46,48,55^. The results of immunoblot analysis were also in accordance with the ELISA results (Fig. 5a–g); SL ECV exhibited the best immuno-recognition compared to other venoms (Fig. 5a–g, Supplementary Figs. S9, S10, S11a,b). In our previous studies, we reported a poor immuno-recognition of low molecular mass (< 20 kDa) Indian snake venom proteins by commercial Indian PAV^6,43–45,49–51^. In this study, however, both batches of SL PAV (compared to Indian PAV) exhibited significantly higher immuno-recognition of low molecular mass proteins of SL snake venoms. Moreover, no batch to batch variations (among B1 and B2) were seen in the immuno-recognition properties of country-specific SL PAV (Fig. 5a–g).

Snake venom enzymes, particularly those from the Viperidae family of snakes, play a vital role in venom-induced toxicity and lethality^45,47,56,57^. Viperidae venoms are rich in enzymatic toxins like phospholipase A_2_ (PLA_2_), snake venom metalloprotease (SVMP), and snake venom serine protease (SVSP), which play an important role in the toxicity of venom^45,47,58–63^. Therefore, assessing the in vitro neutralization of catalytic activities and the pharmacological properties of these enzymes by commercial PAV is another important in vitro method for determining the efficacy of commercial antivenom.

The in vitro neutralization of some enzymatic activities and pharmacological properties of SL snake venoms by country-specific SL PAV, and Indian PAV was compared. The enzymatic activities exhibited by high molecular mass venom proteins (adenosine tri/di/mono phosphatase, l-amino acid oxidase, hyaluronidase, and SVMP) were better neutralized in comparison to low molecular mass venom toxins (e.g., PLA_2_), though the neutralization potency of both batches of SL PAV was higher (p < 0.05) than that of Indian PAVs (Supplementary Fig. S12a–e). Poor neutralization of PLA_2_ activity is a major concern for venom-induced toxicity and lethality^64,65^. Immunoblotting analysis showed that the SDS-PAGE protein band (~ 13–15 kDa) for PLA_2_ is immuno-recognized by the SL PAV; however, the catalytic site of PLA_2_ enzyme, may be due to its poor immunogenicity, is not well neutralized^44^. The enzymatic activity exhibited by HHV was better neutralized by SL PAV than by Indian PAVs since the latter do not contain antibodies against this venom. This result shows a significant improvement in the immuno-recognition and in the neutralization of venom enzymes of SL snake venoms by country-specific SL PAV in comparison to Indian PAV developed against the Indian ‘big four’ snake venoms. In a nutshell, the in vitro laboratory tests provide convincing evidence for the significant improvement of the venom recognition property of the newly developed SL PAV, in comparison to Indian PAV.

### Evaluation of pre-clinical efficacy of country-specific SL PAV

Assessing the neutralization of in vivo lethality and toxicity of snake venoms by commercial PAV is the gold standard for determining their efficacy and it is a crucial step in the clinical assessment of PAV^14,66^. The in vivo venom neutralization potency of SL PAV is shown in Table 2. Recently, the pre-clinical efficacy of a newly developed SL snake venom-specific PAV (raised against SL snake venoms—DRV, ECV, HHV, and NNV) by Instituto Clodomiro Picado (ICP), Costa-Rica, has been assessed^13^. To the best of our knowledge, this PAV has not yet been marketed in SL. Researchers have reported that *B. caeruleus* is responsible for 10–15% of the snakebite in SL^67,68^. ICP PAV does not contain *B. caeruleus* venom in the immunization mixture, therefore this PAV is devoid of antibodies against this medically important snake venom^13^. However, the paraspecific cross-neutralization of *B. caeruleus* venom by antibodies against Elapdiae family of snake venom in ICP PAV may not be ruled out; nevertheless, it cannot ensure full protection to SL krait bite patients^69,70^. Consequently, it may be anticipated that owing to containing antibodies against *B. caeruleus* venom, SL PAV will provide better protection against krait envenomation. In contrast, ICP PAV has a greater lethality neutralization potency against Viperidae venoms (DRV, ECV, and HHV) and a lower neutralization efficacy against NNV, compared to PSVPL SL PAV.

**Table 2:** Determination of lethality of SL venoms (LD_50_ value) and neutralization of lethality (ED_50_ value) of venom samples by two batches of SL PAV.

SL snake venom	LD_50_ value (µg/mouse)	ED_50_ value (mg/mL)		ED_50_ value (µL antivenom required/mg venom)	
		SL PAV B1	SL PAV B2	SL PAV B1	SL PAV B2
*Naja naja*	20.33 ± 1.16	0.732 ± 0.04	0.687 ± 0.06	1400 ± 0.08	1500 ± 0.13
*Bungarus caeruleus*	1.50 ± 0.25	0.523 ± 0.03	0.556 ± 0.06	1900 ± 0.09	1800 ± 0.18
*Daboia russelii*	8.75 ± 0.30	0.786 ± 0.02	0.710 ± 0.04	1300 ± 0.04	1400 ± 0.08
*Echis carinatus*	10.85 ± 0.21	0.586 ± 0.03	0.586 ± 0.02	1700 ± 0.09	1700 ± 0.07
*Hypnale hypnale*	30.19 ± 1.90	0.725 ± 0.06	0.689 ± 0.05	1400 ± 0.12	1500 ± 0.11

Neutralization activity by PAV is expressed as mg venom neutralized per mL of antivenom. The venom stock (1 mg/mL) was used and injected in the mice at different dilution (1:8, 1:10, 1:12.5). The LD_50_ and ED_50_ values given below are means ± SD of five values.

For the treatment against snakebite to be effective and convenient to the clinicians, the neutralization potency of PAV should preferably be adjusted according to the venom yield of snakes. It is further to be mentioned that the neutralization potency of the newly developed SL-specific PAV was based on the average venom yield of prevalent snake species with an intention to assist clinicians of SL who are well versed with the use and dose-regimen of Indian PAV. Hospital management of snakebite with similar dosage of SL-specific PAV compared to Indian PAV (which has been used for snakebite treatment by clinicians in SL for decades and they are well versed with the dosages) would be expected to be easier and more clinically relevant than the currently used Indian PAV. The published report on average venom yields of Sri Lankan species of snakes is not available, however, the same species of snakes in India have reported average venom yields of 330 mg, 22.7 mg, 200 mg and 6.25 mg for NNV, BCV, DRV and ECV, respectively^71^. In another study, the average venom yields have been reported as 126.32 ± 4.31 mg, 8.352 ± 0.64 mg and 75.98 ± 3.66 mg for NNV, BCV and DRV, respectively^72^. Thus, these two reports indicate a wide variation in the average venom yield for the same species of snakes in India probably due to difference in regions of study and/or age and size of the snakes. Further, the amount of venom that can be injected by envenomation into a patient would be expected to be proportional to the venom content (yield) of the species of snake. Therefore, the neutralization potency of PAV against DRV, HHV, and NNV should be higher than that against ECV and BCV, because the venom yield per bite of these two snakes should therefore be lower than the former three species of snakes^73–77^. However, after the product (SL PAV) registration in SL further clinical trials may be undertaken for the effective clinical dose adjustment, if necessary.

Envenomation by the Elapidae family of snakes, such as the mambas (*Dendroaspis* sp.), cobras (*Naja* sp.), and kraits (*Bungarus* sp.) induces a neurotoxic effect that is the primary cause of lethality; albeit, elapids also contain cytotoxins in their venom which lead to significant tissue necrosis^78,79^. In this case, the neutralization of lethality would be an ideal model for assessing the pre-clinical efficacy of antivenoms. Nevertheless, the Viperidae snakes show a wide range of pathophysiological effects such as myonecrosis, dermonecrosis, hemorrhage, edema, coagulopathies, bleeding in various organs, hemodynamic disturbances, and renal disturbances^66,80,81^. These effects are cumulatively responsible for the venom-induced lethality. Therefore, in the case of Viperidae venoms, pre-clinical evaluation of antivenoms by assessing the neutralization of lethality is not sufficient for an integrated evaluation of the antivenoms^66^. The WHO has recommended assessing the neutralization of other toxic activities with supplementary tests and the essential test of neutralization of lethality prior to marketing new antivenoms or introducing existing antivenoms into new geographical locales^14^.

The in vivo neutralization of venom-induced toxicity by the newly developed SL PAV has been determined in mice. Moreover, the toxicities of SL snake venoms are summarized in Supplementary Table S2. The tested pharmacological activities (i.e., hemorrhagic, necrotizing, pro-coagulant, defibrinogenating, and myotoxicity) of DRV, ECV, and HHV are well neutralized by SL PAV with variable potencies (Table 3, Supplementary Fig. S13). The neutralization potency of SL PAV towards hemorrhagic activity, necrotizing activity, and defibrinogenating activity was found to be higher against DRV, in comparison to ECV and HHV. In contrast, the pro-coagulant activity displayed by HHV venom was better neutralized by SL PAV, compared to neutralization of the same activity against DRV and ECV (Table 3). This may be due to the HHV showing much less pro-coagulant activity compared to the other two Viperidae snake venoms (Supplementary Table S2).

**Table 3:** Neutralization of in vivo pharmacological activities of SL snake venoms by newly developed SL PAV.

Pharmacological properties	Neutralization of pharmacological properties [neutralization of toxicity of SL venom (mg)/mL of PAV] (n = 5)				
	*Naja naja*	*Bungarus caeruleus*	*Daboia russelii*	*Echis carinatus*	*Hypnale hypnale*
MHD_50_^a^	NA	NA	24.7* ± 0.7	1.2 ± 0.1	1.1 ± 0.1
MND_50_^b^	NA	NA	40.6* ± 0.3	0.7 ± 0.07	2.8 ± 0.1
MCD-P_100_^c^	NA	NA	NA	3.8 ± 0.5	70.0* ± 11.8
MCD-F_100_^d^	NA	NA	NA	4.1 ± 0.9	36.8* ± 7.4
MDD_100_^e^	NA	NA	2.4 ± 0.4	0.9 ± 0.2	0.2 ± 0.04
MMD_50_^f^	2.3 ± 0.5	2.1 ± 0.4	2.2 ± 0.5	1.4 ± 0.3	3.7 ± 0.7

Values are means ± SD of five values.

*Significance of difference of neutralizaton potency between *D. russelii, E. carinatus* and *H. hypnale* (p < 0.05).

The PAV was dissolved in 10 mL of sterilized water. Neutralization of pharmacological activity is expressed as activity of venom (mg) neutralized per mL of reconstituted PAV in water.

NA indicates no activity by the venom.

^a^Minimum hemorrhagic dose (MHD) is defined as the amount of venom (in μg dry weight) which, when injected intradermally, induces in mice a 10 mm hemorrhagic lesion after a predefined time interval, usually 2–3 h, post injection.

^b^Minimum necrotizing dose (MND) is defined as the smallest amount of venom (in μg dry weight) which, when injected intradermally into anaesthetized mice, results in a necrotic lesion of 5 mm diameter post 3 days of treatment.

^c^The minimum coagulant dose on plasma (MCD-P) is defined as the smallest amount of venom (in mg dry weight per liter of test solution or μg/mL) that induces clotting of citrated human plasma under the experimental conditions.

^d^The minimum coagulant dose on fibrinogen (MCD-F) is defined as the smallest amount of venom (in mg dry weight per liter of test solution or μg/mL) that clots a solution of bovine fibrinogen in 60 s at 37 °C.

^e^Minimum defibrinogenating dose (MDD) is defined as the minimum dose of venom that produces incoagulable blood in all mice within 1 h of intravenous injection.

^f^Mimimum myotoxic dose (MDD) is characterized by the appearance of myoglobin in urine and by increments in the serum levels of muscle-derived enzymes, creatine kinase (CK).

In summary, grave concerns have been expressed by clinicians about using Indian PAVs for the treatment of snakebite in SL as it often shows only partial effectiveness in neutralizing the venom toxicity and it produces adverse clinical reactions in patients. Consequently, because of the long-standing demand for a country-specific PAV for treating snakebite, the PAV was developed against the venoms of five most medically important snakes of SL. The purity of the preparation and the safety of the newly developed SL PAV was found to be satisfactory and no significant variation was seen in the PAV composition or potency between two batches (B1 and B2) of SL PAV; however, for an affirmative conclusion regarding batch-to-batch variation, future studies with more number of PAV batches are warranted. However, following the good manufacturing practice (GMP) by the antivenoms manufacturers can result in production of different batches of PAV with uniform potency and composition^15^. Further, the immunological cross-reactivity studies and enzyme neutralization assay documented the superiority of SL PAV in comparison to Indian PAVs against SL venoms. The pre-clinical study also provided convincing evidence for the neutralization of lethality and toxicity of SL snake venoms by SL PAV. Therefore, the findings of this study illustrate that the improved neutralization potency of PAV against SL snake venoms will greatly augment the hospital management of snake envenomation in SL. Further pre-clinical and clinical studies are still warranted to understand the complex pharmacokinetics and pharmacodynamics, and the venom-antivenom interactions in vivo that can neutralize the venom-induced toxicity.

## Materials and methods

### Venoms and PAV

The following five snake venoms (pooled from 5 to 6 snakes) from SL origin were used in this study: (i) *Naja naja* (NNV), (ii) *Daboia russelii* (DRV), (iii) *Bungarus caeruleus* (BCV), (iv) *Echis carinatus* (ECV), and (v) *Hypnale hypnale* (HHV). The venoms were collected from snakes of either sex of different age from various locations around Kandy city in SL as per prevalence of snakes in that area. The Saw scaled vipers (ECV) were collected from North SL (Jaffna). The collected venoms were stored lyophilized, and sent by University of Peradeniya (SL) who are collaborating with Premium Serums and Vaccines Pvt. Ltd. The equine PAV developed against SL snakes was obtained from Premium Serums and Vaccines Pvt. Ltd. (PSVPL), India [Batch-1 (B1): Batch no—ASVS-SL/LY-001; manufacturing date: April 2019; expiry date: March 2023, Batch-2 (B2): Batch no—ASVS-SL/LY-002; manufacturing date: April 2019; expiry date: March 2023]. The PAVs produced against the ‘big four’ venomous snakes of India were obtained from PSVPL, India (Batch no-ASVS (I) LY-014; expiry date: December 2022), VINS Bioproducts Ltd. (VINS), India (Batch no. 01AS18026, expiry date: April, 2022), Bharat Serums and Vaccines Ltd. (BSVL), India (Batch no. A05319007, expiry date: October 2022). All of the PAVs used in this study contained pepsin digested and caprylic acid purified IgG [F(ab′)_2_]. Affinity-purified horse F(ab′)_2_ and horse IgG were purchased from Jackson Immuno Research Inc, USA and BioRad, USA, respectively. Anti-goat erythrocyte polyclonal antibody (raised in rabbit) was obtained from Fitzgerald Industries International, USA. The Pierce LAL Chromogenic Endotoxin Quantitation Kit was from Thermo Scientific, USA. The NHS-activated Sepharose 4 Fast Flow matrix was purchased from GE Healthcare. All other chemicals were of analytical grade and obtained from Sigma-Aldrich, USA. Human embryonic kidney 293 cells (HEK-293T), murine hepatoma Hepa 1–6 cell line (Hepa1-6), and differentiated rat skeletal (L6) myoblast cell lines were procured from ATCC (American type culture collection), USA.

### Characterization of physicochemical properties of SL PAV

The texture, colour, and homogeneity of the PAV preparation were determined by visual inspection. The examination of textures like macroscopic collapse, color uniformity, cake shrinkage formation and adhesion properties of powder to the vials of PAVs was done by visual inspection and photographs of vials were also captured^16^. PAVs were dissolved in 10 mL of sterilized de-ionized water (provided along with the antivenom) and the turbidity of the solution was assessed by a turbidimeter (model-CL52-D, Nephelometer, ELICO Ltd., India)^16^. The 2 mL PAV solutions were transferred to pre-weighed microfuge tubes and after centrifugation of the tubes at 10,000 rpm for 10 min, the solutions were decanted, and the tubes were dried in vacuum and weighed again to determine the presence of the insoluble component, if any. The pH of the aqueous PAV solution was determined using a digital pH meter (Eutech Instruments, pH 510, USA). For determining the residual moisture content, a measured amount of PAV was heated at 105 °C for 3 h in an oven and moisture content was determined by heat drying method^82^.

### Determination of purity of active substance in SL PAV

#### FPLC gel filtration chromatography method followed by SDS-PAGE analysis

The protein content of PAV was determined as described by Lowry et al.^83^ and the total protein concentration (in g/dL) post dissolving the PAV content in 10 mL of water was determined^19^. The homogeneity of the preparation was determined by size exclusion chromatography of a 50 mg/mL solution of PAV dissolved in 20 mM Tris–HCl containing 150 mM NaCl, pH 7.4 (equilibration buffer) at 4 °C as described previously^19,26,84^. The size exclusion protein peaks and the crude PAV along with horse IgG and its pepsin digested product F(ab′)_2_ were separated by 12.5% SDS-PAGE under reduced and non-reduced conditions^85^. Protein bands were visualized by Coomassie Brilliant Blue R-250 staining. After scanning the gel, intensities of the protein bands were determined by ImageQuant TL 8.1 software (GE Healthcare, Sweden)^19^. Considering that the molecular mass of F(ab′)_2_ is 100 kDa, the F(ab′)_2_ aggregate content of the PAVs was also analyzed by densitometry analyses of the SDS-PAGE protein bands of a mass greater than 100 kDa^28^. The percent of aggregate content (band intensities above 100 kDa) in a particular batch of SL PAV was determined from the cumulative band intensity for that particular batch of PAV.

#### Tandem mass spectrometry analysis to determine the purity of active substance and presence of other contaminating proteins in SL PAV

The procedures described in this section is adopted from our previous publications^19,28^. Hundred microgram proteins of PAV batches were subjected to reduction (10 mM DTT), alkylation (55 mM iodoacetamide) and then digested with trypsin at an enzyme: substrate ratio of 1:30 for overnight (~ 16 h) at 37 °C^45^. The trypsin-digested peptides were desalted and concentrated using ZipTip C18 tips following the manufacturer’s protocol.

The tryptic fragments of PAV were reconstituted in 0.1% formic acid and separated on a Zorbax 300SB-C18 analytical column (75 μm × 150 mm, 3.5 μm, Agilent), coupled to an Agilent 1200 HPLC, at a flow rate of 300 nL/min. The mobile phase gradient for separation of the peptides was set as: 11% B for 5 min, 11 to 25% B for 20 min, 25 to 53% B for 16 min, 53 to 100% B for 5 min, 100% B for 4 min, and then 11% B for 4 min. Solvent A and B were 0.1% formic acid and 80% acetonitrile (ACN) containing 0.1% formic acid, respectively.

The peptides eluted from the HPLC column were then fed into a Nanomate Triversa (Advion BioSciences, Ithaca, NY), equipped with an LC coupler and electrospray ionization (ESI) nanospray chip. The LC coupler connects the flow from the HPLC to the ESI chip, where the nano-ESI generated ions were transferred into an LTQ Orbitrap Discovery hybrid mass spectrometer (Thermo Fisher Scientific, Bremen, Germany). The ionization voltage was set to 1.7 kV and the raw data were acquired in a data-dependent acquisition (DDA) mode by Xcalibur software (Thermo Fisher Scientific, Bremen, Germany). One MS survey scan was followed by 5 MS/MS scans with exclusion duration of 120.0 s during DDA. Survey full-scan MS spectra (from m/z 300–2000 with lock mass set to 445.12 corresponding to polysiloxane) were acquired in Fourier Transform (FT) mode with a resolution of 30,000 (full width at half-maximum). Subsequent fragmentation (MS/MS) was collision-induced dissociation (CID) with normalized collision energy set to 35% in linear ion trap mode. The MS/MS triggering conditions were minimum signal intensity of 10 000; charge state of + 2, + 3; maximum injection time for MS/MS was 500 ms; and isolation width was 2 amu^45^.

The raw LC–MS/MS data were processed to generate peak list in Mascot generic format (*.mgf) using ProteoWizard release version 3.0.331. The peak lists were then searched against the *Equus caballus* (taxid 9796) protein entries of the UniProt database (version 2019_07) using the Mascot search engine (Matrix Science, London, UK, version: 2.4). The following search parameters were used; enzyme: trypsin, maximum missed cleavage sites: 2, precursor ion mass tolerance: 10 ppm, fragment ion tolerance: 0.8 Da, fixed modification: carbamidomethylation on cysteine, variable modifications: oxidation on methionine residues^19,45^.

The relative abundances of the identified proteins were calculated using Exponentially Modified Protein Abundance Index (emPAI)-based label-free quantification technique^86^. The MassSorter v3.1 software (http://services.cbu.uib.no/software/massSorter/downloadMassSorterNew) was used for in silico generation of the observable peptides of the identified proteins^19^.

The relative abundance of a protein (X) in PAV was calculated using the following equation:

$$Relative \; \; abundance \; \; of \; \; X =\frac{emPAI \; \; of \; \; X}{\Sigma \; \;emPAI \; \;of \; \; all \; \; proteins}$$

### Characterization of in vitro safety profile of SL PAV

#### Determination of Fc content in the IgG molecule and assessment of complement activation

The incomplete pepsin digestion of IgG (Fc content of IgG) in the PAV preparation, if any, was determined by ELISA and Western blot analysis using HRP (horseradish peroxidase)-conjugated rabbit anti-horse IgG Fc specific antibody (Sigma Aldrich, USA)^28^. For the ELISA, 100 ng of PAV protein (in triplicate) or BSA (bovine serum albumin) (negative control) or PBS (reagent control) was coated in the 96 wells of the microtiter plate for overnight incubation (~ 18 h) at 4 °C. After blocking the wells with 5% fat-free skimmed milk, 100 µL of anti-horse IgG Fc specific antibody (1:2000 dilution) or PBS (control) was added and the color was developed by adding TMB (3,3′,5,5′-tetramethylbenzidine)/H_2_O_2_ as the substrate^44^. Absorbance was measured at 492 nm in a microplate reader (Multiskan GO, Thermo Scientific, USA). A standard curve of the graded concentrations of purified horse IgG was prepared and Fc content was determined by ELISA under identical experimental conditions. The Fc content of test samples was compared to the standard curve of horse IgG.

For the immunoblot analysis, 20 µg of PAV protein (in triplicate) and horse IgG after separation by 12.5% SDS-PAGE (reduced) was transferred to a PVDF membrane in a semi-dry blotter (Amersham Bioscience, Sweden). After blocking the protein unbound portion of the membrane (non-specific binding sites) by 5% fat-free skimmed milk, it was thoroughly washed with PBS-T in an incubator shaker and the membrane was incubated with HRP-conjugated anti-horse rabbit IgG Fc specific antibody (1:2000 dilution) for 1 h at room temperature. After washing, the membrane blot was developed using the TMB/H_2_O_2_ substrate kit^44^. The image was photographed, scanned (Epson image scanner, Epson America, Inc) and the densitometry analysis of the developed blot was processed with ImageQuant TL 8.1 software (GE Healthcare, Sweden). The percent content of Fc in the purified horse IgG was considered as 100% and the other values were compared to that^28^.

Classical and alternative pathways of complement activation were determined by percentage of hemolysis induced by SL PAV (batch 1 and batch 2) samples^37^. Human blood samples from healthy donor were collected without anticoagulant and allowed to clot at room temperature for 4 h. The collection of blood from healthy volunteers (who were not under medication) was approved by the Tezpur University Ethical Committee and informed consent was obtained from all participants (TU/TUEC/59/08/4017). The serum was separated by centrifugation of blood at 1000×*g* for 10 min and the normal human serum (NHS) was stored at − 80 °C until further use. Goat blood obtained from slaughter house was collected in 3.8% tri-sodium citrate (9:1 ratio) and the blood was centrifuged at 4300 rpm for 10 min at 4 °C to pellet down the erythrocytes^43^.

For determination of complement activation by classical pathway (CP) goat erythrocytes were washed with 1× PBS, pH 7.4 for three times, re-suspended in the same buffer and then incubation with anti-goat erythrocyte antibodies (1:500 dilutions) at 37 °C for 15 min. The antibody-sensitized goat erythrocytes were washed with 1× PBS pH 7.4 buffer and 2% (v/v) erythrocytes suspension was prepared in veronal buffered saline (VBS^2+^—10 mM Na-barbitone, 0.15 mM CaCl_2_ and 0.5 mM MgCl_2_, pH 7.4).

For alternative pathway (AP) of complement activation analysis, goat erythrocytes were washed with 1× PBS, pH 7.4 and re-suspended in AP buffer (10 mM Na-barbitone, 10 mM EGTA and 0.5 mM MgCl_2_; pH 7.4) at a final concentration of 2% (v/v). Fifty micro liters of antibody sensitized goat erythrocytes (for classical pathway) or goat erythrocytes (for alternative pathway) were added to wells of a 96-well microtiter plate containing 50 µL of human serum pre-incubated with 50 µl of SL PAV (1 mg) at 37 °C. The mixture (final volume 150 µL) was incubated for 30 min at 37 °C and then 150 µL of VBS^2+^ was added to each well. The plate was centrifuged at 1300 rpm at 4 °C for 5 min (Heraeus multifuge X1R, Thermo Scientific, USA) and 50 µL of supernatant from each well was transferred to a new 96-well plate containing 200 µL of water. The contents were mixed well by shaking, and the absorbance of mixture was measured at 414 nm in a microplate reader. The results were expressed as the percentage of CH50/mL (classical pathway) or AP50/mL (alternative pathway) activation compared with NHS incubated with saline (100%).

#### Determination of IgA and IgE contamination

The occurrence of IgA and IgE in the SL PAVs, if any, was determined by Western blot analysis and ELISA of PAV against HRP-conjugated anti-horse IgA and anti-horse IgE antibodies, respectively (1:2000 dilution) as described previously^19,28^. As a negative control, 20 µg (for the Western blot analysis) or 100 ng (for the ELISA) of BSA was used. The blots were scanned (EPSON image scanner, Epson America, Inc.) and their densitometry analysis was done by ImageQuant TL 8.1 software (GE Healthcare, Sweden).

#### Sterility test and determination of endotoxin level

The sterility of the SL PAV was tested according to the WHO guidelines^14^. One mg of PAV solution in sterile water was incubated in trypticase soy broth and thioglycolate medium. Control culture flasks were included for each medium. Flasks were incubated at 25 °C or at 35 °C for 14 days in trypticase soy broth and thioglycolate medium to test for fungal and bacterial cultures, respectively, using the appropriate controls. Culture flasks were examined daily for bacterial or fungal growth by checking the optical density in a spectrophotometer at 600 nm.

The level of endotoxin contamination in SL PAV, if any, was determined using a commercial diagnostic kit (Pierce LAL Chromogenic Endotoxin Quantitation Kit, Thermo Scientific, USA)^28^. Briefly, a standard curve of graded concentrations of *E. coli* endotoxin was plotted so that the endotoxin concentration in test sample can be determined as low as 0.1 EU/mL. For endotoxin determination, 50 µL of SL PAV samples in triplicate was added to wells of microtiter plate and the plates were incubated at 37 °C for 5 min at dark. Then, 50 µL of Limulus amebocyte lysate (LAL) to each well was added, mixed gently on a plate shaker for 10 s followed by incubation at 37 °C for 10 min. Thereafter 100 µL of chromogenic substrate solution was added to each well and incubated for 6 min at 37 °C. The reaction was stopped by adding 50 µL of 25% acetic acid and the absorbance of mixture was read at 405 nm in a microplate reader (Multiskan GO, Thermo Scientific, USA). From the standard curve, the concentration of endotoxin in PAV samples was determined. As a negative control, BSA was used.

#### Determination of preservative content

The use of a specified amount of preservative (*m*-cresol) in PAV preparations for their long-term storage was approved by the WHO in 2016^14^. The *m*-cresol content was determined by reversed-phase ultra-high performance liquid chromatography (RP-UHPLC) of SL PAV on an Acclaim 300 C_18_ RP-UHPLC column (2.1 × 150 mm, 3 µm) pre-equilibrated with 60% methanol in 0.1% (v/v) trifluoroacetic acid (TFA) as described previously^19,28^. The flow rate was 0.5 mL/min and the column temperature was maintained at 30 °C. The isocratic programme for the mobile phase was optimized for 18 min^87^. The detection of *m*-cresol was observed at 254 nm^88^. The percentage of *m*-cresol was determined against a standard curve of *m*-cresol run in UHPLC under identical experimental conditions.

#### Assessment of in vitro hemolytic activity, and cytotoxicity against mammalian cells

The in vitro direct and indirect hemolytic activity of SL PAV (20 μg/mL) was determined against washed human erythrocytes^89,90^. The in vitro cell cytotoxicity was determined by incubating 10 μg/mL of PAV or PBS (control) against human embryonic kidney 293 (HEK-293T), murine hepatoma Hepa 1–6 (Hepa1-6), and differentiated rat skeletal (L6) myoblast cells (1 × 10^5^ cells/mL) grown in DMEM for 72 h at 37 °C and 5% CO_2_^28^. Thereafter, cell viability was determined by the MTT-based method^91^ and the result was expressed as PAV-induced cell death (in percentage), if any, with respect to control (PBS-treated cells)^60^. H_2_O_2_ was used as a cytotoxic agent (positive control).

#### Comparison of SL PAV with Indian PAV in terms of immunological cross-reactivity, and in vitro neutralization of some enzyme activities and pharmacological properties of SL snake venoms

The immunological cross-reactivity of SL snake venoms against SL PAV and Indian PAV raised against venoms of the Big Four snakes of India was determined by ELISA and Western blot analysis^19,43–45^. Briefly, for ELISA 100 ng of venom was coated for overnight at 4 °C in microtiter ELISA plate. After washing the wells by using washing buffer (phosphate buffer saline containing 0.5% tween-20) the wells were blocked by 5% skimmed milk. The SL PAV or Indian PAV (1 mg/mL) were used as primary antibody (1:500 dilutions) and incubated for 2 h with venom or BSA (blank control) at room temperature. For negative control, the venom samples were treated with naïve horse IgG and developed in parallel. After incubation with primary antibody, the excess antibodies were washed using washing buffer and incubated with anti-horse IgG HRP-conjugated secondary antibody (produced in rabbit) for 2 h at room temperature (1:2000 dilutions). The wells are washed properly and TMB/H_2_O_2_ substrate was used for development of color. The reaction was stopped immediately by 2 M H_2_SO_4_ and the absorbance was read at 492 nm. For presenting the data, the absorbance values of PAV against venom samples was deducted from the absorbance of negative control.

Immunoblotting experiments were performed as described previously by resolving the venom proteins in 12.5% SDS-PAGE under reduced conditions^44,45^. The SDS-PAGE protein bands were transferred to PVDF membrane in a semi-dry gel transfer system (Amersham Bioscience, UK) at 1.2 mA/cm^2^ of membrane area for 2 h. The transfer efficiency was checked by Ponceau S staining of the membranes. The membranes were blocked by 5% skimmed milk (prepared in Tris buffered saline with 0.1% Tween-20; TBS-T) for overnight at 4 °C. Thereafter, the membranes were washed with TBS-T and incubated with primary antibody (15 mg/mL PAVs) at 1:1000 dilutions for 1 h at room temperature. The excess unbound antibodies were washed with TBS-T and incubated with anti-horse IgG ALP-conjugated secondary rabbit anti-horse antibodies for 1 h at room temperature. Thereafter, the blots were developed using 5-bromo-4-chloro-3-indolyl-phosphate/nitro blue tetrazolium (BCIP/NBT) kit (Sigma-Aldrich, USA) and scanned (Epson Expression 11000XL, USA). Venom samples treated with horse naïve IgG served as negative control. Densitometry analysis of the blots was done using ImageQuant TL software 8.1 (GE Healthcare, Sweden).

Neutralization of some enzyme activities (ATPase, ADPase, AMPase, l-amino acid oxidase, fibrino(geno)lytic, metalloproteinase, hyaluronidase, phospholipase A_2_) and pharmacological properties (pro/anti-coagulant activity) of SL snake venoms by the newly developed SL PAV and Indian PAVs was determined as described previously^44,45,51,52^. Briefly, venom (10 µg) was pre-incubated with PAV (100 µg) in a predetermined ratio (1:10, protein: protein) for 30 min at 37 °C followed by assaying the mixture for enzymatic activities and in vitro pharmacological properties of venom^44,45,51,52^. The enzymatic activities and pharmacological properties displayed by crude venom were considered as 100% activity and other values were compared to that^19,48^.

### Assessment of pre-clinical efficacy: neutralization of lethality and in vivo pharmacological properties of snake venoms by SL PAV

#### Ethical statement

In vivo neutralization of lethality and other pharmacological effects of snake venom hemorrhagic activity, necrotizing activity, pro-coagulant activity, defibrinogenating activity, and myotoxicity of SL snake venoms by PAV raised against these snakes were evaluated in laboratory inbred Swiss albino mice (males and females) weighing between 18 and 20 g, age 3 to 4 weeks, following the WHO guidelines^14^. Animal experiments were conducted in the animal house facility of Premium Serums & Vaccines Pvt. Ltd, Pune. All animal experiments were done in accordance with the ARRIVE (Animal Research: Reporting of in Vivo Experiments) guidelines and ethical guidelines of the “Committee for the purpose of control and supervision of experiments in animals (CPCSEA)” (the approval numbers for the animal experiments are shown in Supplementary Table S3). Mice were kept in cages in a standard environment with temperature between 22 and 25 °C, relative humidity between 50 and 60%, 12 h daylight cycle and 12–15 air changes per hour. Dry food pellets (Nutritive Life Sciences, Pune) and purified filtered water were provided ad libitum.

#### Determination of in vivo neutralization of lethality

The stock solution of venom (1 mg/mL) was diluted in normal saline to prepare different concentrations of venom solution (0.8-fold decrement) so that the middle dose was expected to be the LD_50_ dose of the venom (where 50% of the test animals died). To determine the LD_50_, graded concentrations of venom from each species of snake (in 5 mL of normal saline) were injected intravenously into a group of five mice. Animals were observed for 48 h and deaths during this period, if any, were recorded. The LD_50_ was calculated by the Reed and Muench method (1938) by using the following formula.

$$\text{Log} \; \text{of} \; \text{LD}50 =[\text{Log} \; \text{of} \; \text{dilution} \; \text{above } \; 50\% \; \mathrm{ mortality}- \frac{[\text{Mortality} \; \text{above } \; 50{\%}-50]}{[\text{Mortality} \; \text{above }50 {\%}-\text{Mortality} \; \text{below } \; 50 {\%}]} \times [\text{Log} \; \text{of} \; \text{decrement}]$$

A fixed amount of venom (“challenge dose”, usually corresponding to 5LD_50_) was mixed with graded concentrations (1.5 fold) of the PAV and adjusted to a constant final volume with saline. The mixtures were incubated for 30 min at 37 °C, and then aliquots of a precise volume (maximum 0.5 mL intravenously) of each mixture were injected into groups of 6 Swiss albino mice. A control group was injected with a mixture of the venom “challenge dose” with saline solution alone (no PAV) to confirm that the venom “challenge dose” induces 50% lethality. After injection, deaths were recorded at 48 h (intravenous test) and the results were analyzed using Reed and Muench method. One antivenom ED_50_ dose is the amount of antivenom, or the venom/PAV ratio, resulting in the survival of 50% of mice injected with a mixture of PAV and a lethal quantity of venom. The ED_50_ results were expressed as mg of venom neutralized by per mL of PAV and value was calculated using following formula^14^.

$$\text{Log} \; \text{of} \; \text{ED}50 =[\text{Log} \; \text{of} \; \text{dilution} \; \text{above } \; 50 \; \%\mathrm{ mortality}- \frac{\left[\text{Mortality} \;\text{above } \; 50{\%}-50\right]}{\left[\text{Mortality} \; \text{above }50{\%}-\text{Mortality} \; \text{below } \; 50 {\%}\right]} \times [\text{Log} \; \text{ of} \; \text{dilution} \; \text{factor}]$$

In addition to the neutralization potency (mg of venom neutralized by 1 mL of PAV) value, a universal unit for comparison of ED_50_ values in terms of µL PAV required/ mg venom were also calculated^92^.

#### Neutralization of in vivo pharmacological activities of snake venoms

To determine the hemorrhagic activity, five different concentrations of venom solutions (50 μL) or normal saline (control) were injected intradermally in the shaved skin of lightly anesthetized mice (n = 5). After 3 h post injection, mice were euthanized using a carbon dioxide asphyxiation method. The area of the injected skin was removed and the size of the hemorrhagic lesion was measured using calipers in two directions. The mean diameter of the hemorrhagic lesion for each venom dose was calculated and the mean lesion diameter was plotted against each venom dose to determine the minimum hemorrhagic dose (MHD). One unit of MHD is defined as the dose of venom that produces 10 mm diameter of skin hemorrhage.

To determine the venom necrotizing activity, the above procedure was followed and the size of the dermonecrotic lesion was measured. The mean diameter of the dermonecrotic lesion for each venom dose was calculated and mean lesion diameter was plotted against venom dose to determine the minimum necrotic dose (MND), which is defined as the venom dose that produces skin necrosis with a diameter of 5 mm.

To determine the in vivo defibrinogenating activity, graded concentrations of venom in 0.2 mL of normal saline or only normal saline (control) were intravenously (i.v.) injected in a group of five mice. After 1 h following the injection, blood was withdrawn by cardiac puncture from the anesthetized mice and transferred to glass tubes. Clot formation was observed visually by the tilting of the tubes. The minimum defibrinogenating dose (MDD) is defined as the amount of injected venom that does not show in vitro blood clot formation.

To determine myotoxic activity, five different doses of venom dissolved in 50 μL of normal saline were injected into the right gastrocnemius muscle in each mouse (n = 5). Control animals received an injection of the same volume of normal saline. Blood was withdrawn from the tail tip of the anesthetized mice 3 h post injection and serum creatine phosphokinase (CPK) activity was determined using a commercial diagnostic kit (Tulip Diagnostic, India)^93,94^.

For the neutralization assay, a challenge dose of venom was incubated with different concentrations of PAV at 37 °C for 30 min and the venom-antivenom mixture was injected into a group of five mice to determine the neutralization of the above mentioned pharmacological activities of venom^14,95^.

#### Statistical analysis

The significance of difference for more than one set of data was analyzed by a one way ANOVA. The significance of difference between two sets of data was determined by Student’s t test, using Sigma Plot 11.0 for Windows (version 10.0). A *p* value of ≤ 0.05 between two sets of data was considered to be statistically significant.

#### Ethics declaration

Authors confirm that all methods were carried out in accordance with relevant guidelines and regulations and informed consent was obtained from all subjects. All the animal experimental protocols were approved by Premium Serum and Vaccines Pvt. Ltd., Pune, India, following the ARRIVE guidelines and guidelines of Committee for the purpose of control and supervision of experiments in animals (CPCSEA). The details regarding ethics committee/PSVPL animal ethical review board approval numbers for animal experiments are mentioned in Supplementary Table S3.

## Supplementary Information

Supplementary Information.

## Data Availability

Authors confirm that all relevant data are included in the paper and/or its supplementary information file.
