# Supplementary material for: Assessment of quality and pre-clinical efficacy of a newly developed polyvalent antivenom against the medically important snakes of Sri Lanka
Source: Sci Rep. 2021 Sep 14;11:18238. doi: 10.1038/s41598-021-97501-2 (PMC8440654; doi:10.1038/s41598-021-97501-2)
Supplement: Supplementary file 1 — Supplementary Information. [file 41598_2021_97501_MOESM1_ESM.docx]

**Supplementary information**

**Assessment of quality and pre-clinical efficacy of a newly developed polyvalent antivenom against the medically important snakes of Sri Lanka**

Aparup Patra^1^, Bhargab Kalita^1^, Milind V. Khadilkar ^2^, Nitin C. Salvi ^2^, Pravin V. Shelke ^2^, Ashis K. Mukherjee ^1,3*^

^1^ Microbial Biotechnology and Protein Research Laboratory, Department of Molecular Biology and Biotechnology, School of Science, Tezpur University, Tezpur- 784028, Assam, India. ^2^ Premium Serums and Vaccines Pvt. Ltd, Narayangaon, Pune- 410504, Maharashtra, India. ^3^ Institute of Advanced Study in Science and Technology, Vigyan Path, Garchuk, Paschim Boragaon, Guwahati 781035, Assam, India. *Corresponding Author: Dr. A.K. Mukherjee, Institute of Advanced Study in Science and Technology, Vigyan Path, Garchuk, Paschim Boragaon, Guwahati 781035, Assam, India Tel: +917896003886; E-mail address: akm@tezu.ernet.in

**Supplementary Table S1:** List of proteins identified by ESI-LC-MS/MS analysis of the two batches of SL PAVs (batch 1 and batch2). The data were searched against *Equus caballus* protein entries in UniProt databases.

| **Accession No.** | **Description** | **Score** | **Mass (Da)** | **Sequence coverage (%)** | **No. of significant sequences** | **emPAI** |
| --- | --- | --- | --- | --- | --- | --- |
| **SL PAV B1** | | | | | | |
| A0A0A1E949 | Immmunoglobulin lambda light chain variable region (Fragment) | 2196 | 23111 | 0.48 | 9 | 5.17 |
| A0A0A1E6N9 | Immmunoglobulin lambda light chain variable region (Fragment) | 1830 | 23065 | 0.52 | 9 | 5.17 |
| A0A0A1E9A8 | Immmunoglobulin lambda light chain variable region (Fragment) | 1812 | 23455 | 0.41 | 9 | 5.01 |
| A0A0A1E406 | Immmunoglobulin lambda light chain variable region (Fragment) | 1736 | 23493 | 0.4 | 8 | 3.89 |
| A0A0A1E470 | Immmunoglobulin lambda light chain variable region (Fragment) | 1719 | 23805 | 0.35 | 8 | 3.82 |
| A0A0A1E6E2 | Immmunoglobulin lambda light chain variable region (Fragment) | 1718 | 23631 | 0.45 | 8 | 3.85 |
| A0A0A1E6Q9 | Immmunoglobulin lambda light chain variable region (Fragment) | 1714 | 23322 | 0.32 | 7 | 3.06 |
| A0A0A1E6R9 | Immmunoglobulin lambda light chain variable region (Fragment) | 1702 | 23710 | 0.32 | 7 | 2.96 |
| A0A0A1E483 | Immmunoglobulin lambda light chain variable region (Fragment) | 1686 | 23753 | 0.45 | 8 | 3.82 |
| A0A0A1E9D7 | Immmunoglobulin lambda light chain variable region (Fragment) | 1506 | 23763 | 0.24 | 6 | 2.25 |
| A0A0A1E3Y3 | Immmunoglobulin lambda light chain variable region (Fragment) | 1322 | 23744 | 0.32 | 6 | 2.25 |
| A0A0A1E993 | Immmunoglobulin lambda light chain variable region (Fragment) | 1209 | 23195 | 0.5 | 7 | 3.09 |
| A0A0A1E6I1 | Immmunoglobulin lambda light chain variable region (Fragment) | 1157 | 23598 | 0.36 | 5 | 1.68 |
| A0A0A1E439 | Immmunoglobulin lambda light chain variable region (Fragment) | 1117 | 23403 | 0.31 | 5 | 1.71 |
| A0A0A1E513 | Immmunoglobulin lambda light chain variable region (Fragment) | 1073 | 23148 | 0.34 | 6 | 2.34 |
| A0A0A1E927 | Immmunoglobulin lambda light chain variable region (Fragment) | 816 | 23039 | 0.46 | 6 | 2.36 |
| A0A0B4J1C4 | Joining chain of multimeric IgA and IgM | 122 | 18297 | 0.21 | 4 | 1.76 |
| A0A3Q2H4P9 | Fibronectin | 4148 | 237286 | 0.32 | 50 | 1.7 |
| A0A3Q2HTG2 | Fibrinogen alpha chain | 1178 | 77864 | 0.22 | 13 | 1.2 |
| F6PH38 | Fibrinogen beta chain | 127 | 56898 | 0.07 | 3 | 0.28 |
| A0A3Q2L7R0 | Plasminogen | 586 | 94612 | 0.19 | 10 | 0.65 |
| F6R942 | Alpha-2-macroglobulin | 530 | 161167 | 0.09 | 8 | 0.3 |
| A0A3Q2H333 | Serum albumin | 522 | 71310 | 0.2 | 10 | 0.94 |
| A0A3Q2HBR4 | Haptoglobin | 371 | 41296 | 0.27 | 8 | 1.48 |
| **SL PAV B2** | |  |  |  |  |  |
| A0A0A1E470 | Immmunoglobulin lambda light chain variable region (Fragment) | 1439 | 23805 | 0.41 | 9 | 4.86 |
| A0A0A1E6Q9 | Immmunoglobulin lambda light chain variable region (Fragment) | 1414 | 23322 | 0.38 | 8 | 3.96 |
| A0A0A1E406 | Immmunoglobulin lambda light chain variable region (Fragment) | 1393 | 23493 | 0.39 | 8 | 3.89 |
| A0A0A1E483 | Immmunoglobulin lambda light chain variable region (Fragment) | 1382 | 23753 | 0.41 | 9 | 4.86 |
| A0A0A1E4T2 | Immmunoglobulin lambda light chain variable region (Fragment) | 1374 | 23585 | 0.41 | 9 | 4.96 |
| A0A0A1E452 | Immmunoglobulin lambda light chain variable region (Fragment) | 1363 | 23568 | 0.36 | 8 | 3.89 |
| A0A0A1E976 | Immmunoglobulin lambda light chain variable region (Fragment) | 1361 | 21815 | 0.38 | 8 | 4.51 |
| A0A0A1E6E2 | Immmunoglobulin lambda light chain variable region (Fragment) | 1361 | 23631 | 0.36 | 8 | 3.85 |
| A0A0A1E9C4 | Immmunoglobulin lambda light chain variable region (Fragment) | 1359 | 23958 | 0.37 | 8 | 3.75 |
| A0A0A1E6R9 | Immmunoglobulin lambda light chain variable region (Fragment) | 1358 | 23710 | 0.36 | 8 | 3.82 |
| A0A0A1E4L2 | Immmunoglobulin lambda light chain variable region (Fragment) | 1356 | 23426 | 0.36 | 8 | 3.93 |
| A0A0A1E3Z9 | Immmunoglobulin lambda light chain variable region (Fragment) | 1355 | 23277 | 0.36 | 8 | 3.96 |
| A0A0A1E9D7 | Immmunoglobulin lambda light chain variable region (Fragment) | 1330 | 23763 | 0.31 | 7 | 2.96 |
| A0A0A1E464 | Immmunoglobulin lambda light chain variable region (Fragment) | 1050 | 23839 | 0.37 | 8 | 3.78 |
| A0A0A1E9A8 | Immmunoglobulin lambda light chain variable region (Fragment) | 1038 | 23455 | 0.41 | 9 | 5.01 |
| A0A0A1E6I7 | Immmunoglobulin lambda light chain variable region (Fragment) | 882 | 23297 | 0.43 | 7 | 3.06 |
| A0A0A1E944 | Immmunoglobulin lambda light chain variable region (Fragment) | 839 | 23947 | 0.33 | 7 | 2.91 |
| A0A0A1E3Y3 | Immmunoglobulin lambda light chain variable region (Fragment) | 823 | 23744 | 0.33 | 7 | 2.96 |
| A0A0A1E439 | Immmunoglobulin lambda light chain variable region (Fragment) | 799 | 23403 | 0.31 | 5 | 1.71 |
| A0A0A1E513 | Immmunoglobulin lambda light chain variable region (Fragment) | 783 | 23148 | 0.34 | 6 | 2.34 |
| A0A0A1E998 | Immmunoglobulin lambda light chain variable region (Fragment) | 751 | 23213 | 0.28 | 5 | 1.73 |
| A0A0A1E6F7 | Immmunoglobulin lambda light chain variable region (Fragment) | 749 | 23567 | 0.28 | 5 | 1.7 |
| A0A0A1E550 | Immmunoglobulin lambda light chain variable region (Fragment) | 748 | 23633 | 0.28 | 5 | 1.68 |
| A0A0A1E3W9 | Immmunoglobulin lambda light chain variable region (Fragment) | 559 | 23367 | 0.24 | 4 | 1.22 |
| A0A3Q2HTG2 | Fibrinogen alpha chain | 531 | 77864 | 0.13 | 5 | 0.35 |
| A0A3Q2H4P9 | Fibronectin | 2050 | 237286 | 0.17 | 25 | 0.64 |
| A0A3Q2H333 | Serum albumin | 235 | 71310 | 0.15 | 7 | 0.59 |
| A0A3Q2HBR4 | Haptoglobin | 209 | 41296 | 0.18 | 4 | 0.58 |
| F6R942 | Alpha-2-macroglobulin | 154 | 161167 | 0.02 | 2 | 0.09 |
| A0A3Q2I464 | Prothrombin | 67 | 71509 | 0.01 | 1 | 0.07 |
| A0A3Q2L7R0 | Plasminogen | 117 | 94612 | 0.06 | 3 | 0.16 |

**Supplementary Table S2:** Pharmacological and enzymatic activities of Sri Lanka snake venoms. Values are mean ± SD of five experiments

| **Toxic activity** | **Sri Lanka snake venoms** | | | | |
| --- | --- | --- | --- | --- | --- |
|  | ***N. naja*** | ***B. caeruleus*** | ***D. russelii*** | ***E. carinatus*** | ***H. hypnale*** |
| Haemorrhagic activity, MHD^a^ value (µg/mouse) | No activity up to 40 µg dose, mice died with 50 µg dose | No activity up to 20 µg dose, mice died with 30 µg dose | 5.1 + 0.1 | 1.1+ 0.1 | 4.1 + 0.2 |
| ecrotizing activity, MND^b^ value (µg/mouse) | No activity up to 40 µg dose, mice died with 50 µg dose | No activity up to 20 µg dose, mice died with 30 µg dose | 60.84 + 0.48 | 2.0 + 0.1 | 20.5 + 0.3 |
| Pro-coagulant activity on plasma, MCD-P^c^ value (µg/mL) | No coagulation observed till 10 mg/mL | No coagulation observed till 10 mg/mL | No coagulation observed till 10 mg/mL | 6.3 + 0.6 | 51.7 + 2.89 |
| Pro-coagulant activity on fibrinogen, MCD-F^d^ on value (µg/mL) | No coagulation observed till 10 mg/mL | No coagulation observed till 10 mg/mL | No coagulation observed till 10 mg/mL | 15.3 + 0.6 | 60.7 + 1.1 |
| De-fibrinogenating activity, MDD^e^ value (µg/mL) | No activity up to 25 µg dose, mice died with 30 µg dose | No activity up to 2.0 µg dose, mice died with 2.5 µg dose | 7.60 + 0.55 | 0.6 + 0.1 | 3.6 + 0.5 |
| Myotoxic activity, MMD^f^ value (µg/mouse) | 11.6 ++ 0.9 | 10.8 ++ 1.10 | 10.40 ++ 0.89 | 4.8 ++ 1.1 | 41.6 ++ 1.7 |

^a^ Minimum haemorrhagic dose (MHD) of a venom defined as the amount of venom (in μg dry weight) when injected intradermally, induces a 10 mm haemorrhagic lesion in mice after a predefined time interval, usually 2–3 hours, after injection

^b^ Minimum necrotizing dose (MND) of a venom is defined as the smallest amount of venom (in μg dry weight) when injected intradermally into anaesthetized mice, results in a necrotic lesion of 5 mm diameter post 3 days treatment.

^c^ The minimum coagulant dose on plasma (MCD-P) is defined as the smallest amount of venom (in mg dry weight per litre of test solution or μg/mL) that clots citrated human plasma under the same conditions as compared to control.

^d^ The minimum coagulant dose on fibrinogen (MCD-F) is defined as the smallest amount of venom (in mg dry weight per litre of test solution or μg/mL) that clots a solution of bovine fibrinogen in 60 seconds at 37°C.

^e^ Minimum de-fibrinogenating dose (MDD) is defined as the minimum dose of venom that produces incoagulable blood in all mice tested within 1 hour of intravenous injection.

^f^ Mimimum myotoxicity dose (MDD)is characterized by the appearance of myoglobin in urine and by increase in the serum levels of muscle-derived creatine kinase (CK) enzyme.

**Supplementary Table S3:** Reference numbers of the approvals for the animal experiments.

| **Sr. No.** | **Title of the Project** | **Protocol No.** | **Venoms Studied** |
| --- | --- | --- | --- |
| 1 | Toxicity findings of medically important Sri –Lankan venoms by LD_50_ method | LD50-M / 01/19 | *Naja naja (SL)*  *Daboia russelii (SL)*  *Bungarus caeruleus (SL)*  *Echis carinatus (SL)*  *Hypnale hypnale (SL)* |
| 2 | To determine Minimum Haemorrhagic dose (MHD) of venoms of medically important Sri –Lankan snakes | MHD-M / 01/19 | *Naja naja (SL)*  *Daboia russelii (SL)*  *Bungarus caeruleus (SL)*  *Echis carinatus (SL)*  *Hypnale hypnale (SL)* |
| 3 | To determine Minimum Necrotizing dose (MND) of venoms of medically important Sri –Lankan snakes | MND-M/ 01/19 | *Naja naja (SL)*  *Daboia russelii (SL)*  *Bungarus caeruleus (SL)*  *Echis carinatus (SL)*  *Hypnale hypnale (SL)* |
| 4 | To determine Minimum Defibrinogenating Dose (MDD) of venoms of medically important Sri –Lankan snakes | MDD-M/ 01/19 | *Naja naja (SL)*  *Daboia russelii (SL)*  *Bungarus caeruleus (SL)*  *Echis carinatus (SL)*  *Hypnale hypnale (SL)* |
| 5 | To determine Minimum Myotoxic Dose (MMD) of venoms of medically important Sri –Lankan snakes | MMD-M/ 01/19 | *Naja naja (SL)*  *Daboia russelii (SL)*  *Bungarus caerulus (SL)*  *Echis carinatus (SL)*  *Hypnale hypnale(SL)* |
| 6 | To determine neutralizing effective dose of Snake Venom AntiSerum prepared by PSVPL against Sri Lankan Venoms by using ED50 method | ASVS –SL (PSVPL) Vs SL-V/ED50 -M /01/19 | *Naja naja (SL)*  *Daboia russelii (SL)*  *Bungarus caeruleus (SL)*  *Echis carinatus (SL)*  *Hypnale hypnale (SL)* |
| 7 | To determine neutralizing Minimum Haemorrhagic Dose (MHD) – effective dose of Snake Venom AntiSerum prepared by PSVPL against Sri Lankan Venoms by using ED50 method | ASVS –SL (PSVPL) Vs SL-V/ED50 - MHD -M /01/19 | *Naja naja (SL)*  *Daboia russelii (SL)*  *Bungarus caeruleus (SL)*  *Echis carinatus (SL)*  *Hypnale hypnale (SL)* |
| 8 | To determine neutralizing Minimum Necrotizing Dose (MND) – effective dose of Snake Venom AntiSerum prepared by PSVPL against Sri Lankan Venoms Venoms by using ED50 method | ASVS –SL (PSVPL) Vs SL-V/ED50 - MND -M /01/19 | *Naja naja (SL)*  *Daboia russelii (SL)*  *Bungarus caeruleus (SL)*  *Echis carinatus (SL)*  *Hypnale hypnale (SL)* |
| 9 | To determine neutralizing Minimum Defibrinogenating Dose (MDD) – effective dose of Snake Venom AntiSerum prepared by PSVPL against Sri Lankan Venoms by using ED_50_ method | ASVS –SL (PSVPL) Vs SL-V/ED50 - MDD -M /01/19 | *Naja naja (SL)*  *Daboia russelii (SL)*  *Bungarus caeruleus (SL)*  *Echis carinatus (SL)*  *Hypnale hypnale (SL)* |
| 10 | To determine neutralizing Minimum Myotoxic Dose (MMD) – effective dose of Snake Venom AntiSerum prepared by PSVPL against Sri Lankan Venoms by using ED_50_ method | ASVS –SL (PSVPL) Vs SL-V/ED50 - MMD -M /01/19 | *Naja naja (SL)*  *Daboia russelii (SL)*  *Bungarus caerulus (SL)*  *Echis carinatus (SL)*  *Hypnale hypnale(SL)* |


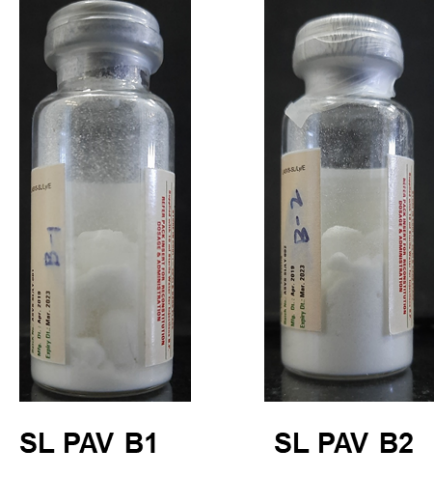


**Supplementary Fig. S1.** Physical appearance of SL PAVs batch 1 (B1) and batch (B2). SL PAV B1 showed more fragmented cake like structure than SL PAV B2. Images are captured by using digital camera (Nikon D5300).


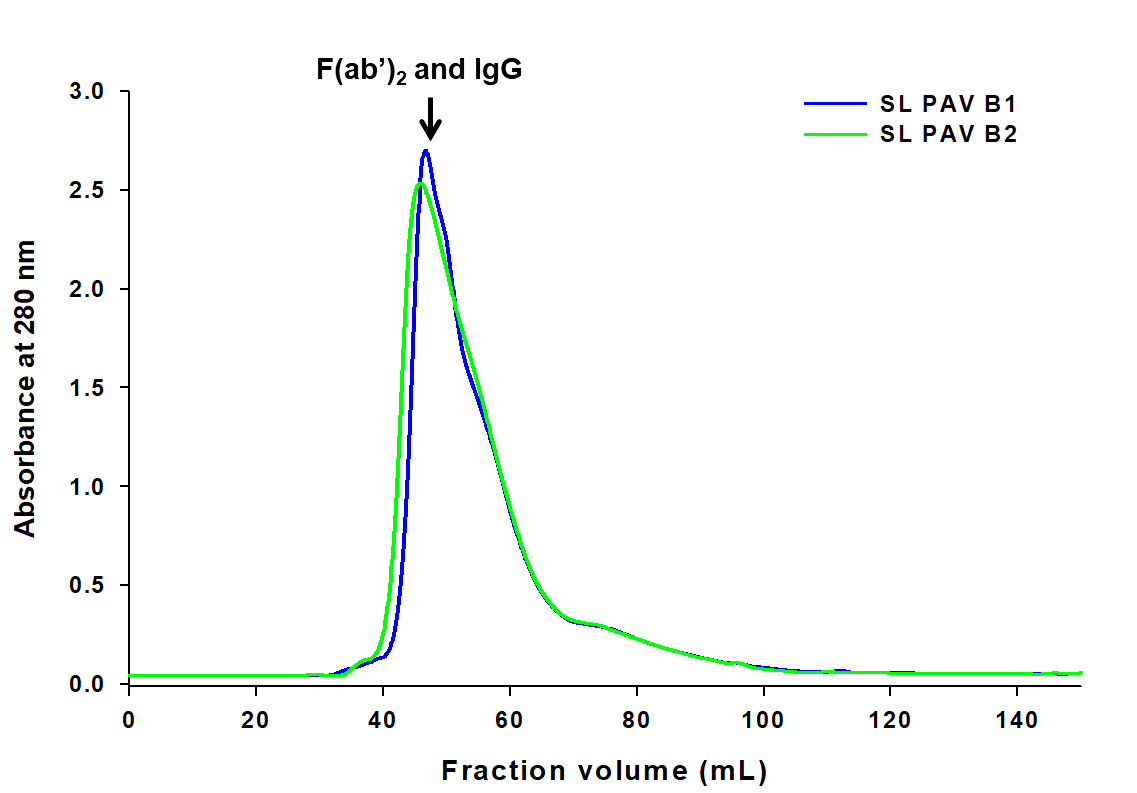


**Supplementary Fig S2.** Gel filtration chromatography of two batches of SL PAV on a Sephacryl S-200 gel filtration column (60 cm × 16 mm; column volume-120 mL) coupled to the FPLC system. The fractionation procedure is described in the text. Satellite figure represents fractionation of purified horse IgG obtained from BioRad, USA and F(ab’)_2_ obtained from Jackson ImmunoResearch Inc, USA, (5 mg dry weight) in identical conditions. The figure is reproduced with due permission from Patra et al., 2018.^1^

**Supplementary Fig S3.** Analysis of SL PAV on the complement activation pathways. Values are mean ± SD of triplicate determinations. No significant difference (p>0.05) was observed between two batches of SL PAV.


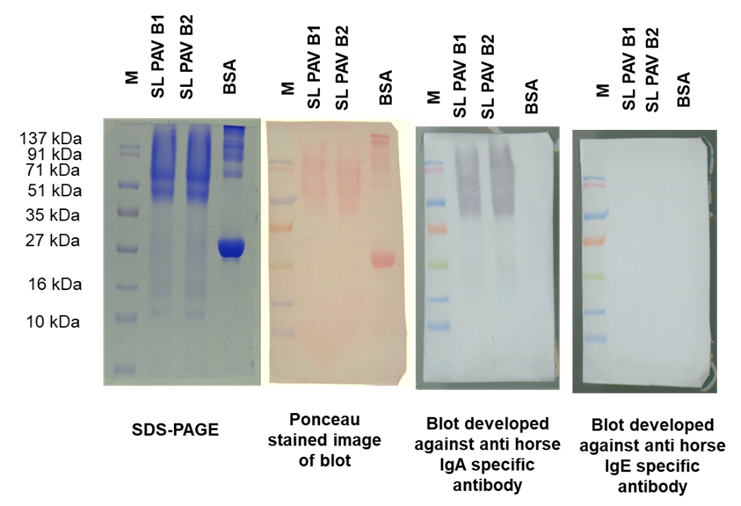


**b**

**a**

**c**

**Supplementary Fig S4.** Immunological cross-reactivity between two batches of SL PAV and anti-horse IgA antibodies (HRP Conjugated) by **(a)** ELISA, and **(b)** western blot analysis followed by **(c)** densitometry analysis of antibody protein recognized bands. Values are mean ± SD of triplicate determinations. No significant difference (p>0.05) was observed between two batches of SL PAV.

**
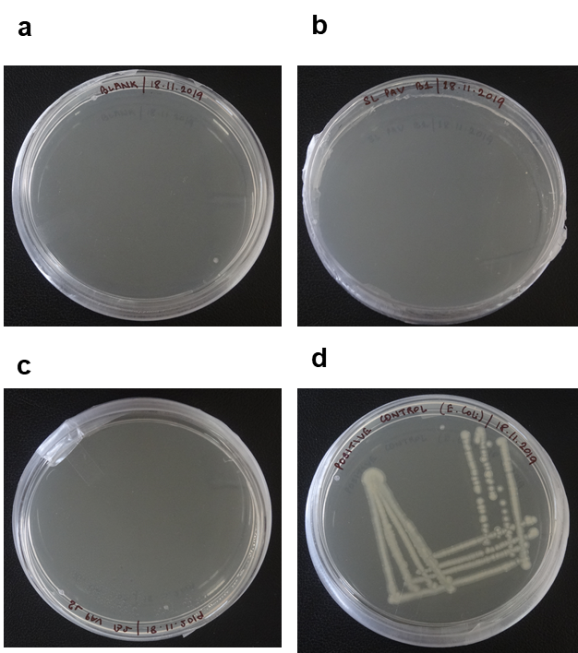
**

**Supplementary Fig S5.** Determination of microbial contamination in the SL PAVs. The antivenoms are incubated with LB broth culture media with positive (*Escherichia* *coli*) and negative control (sterile water). After 24 h of incubation the culture was plated in agar plate and visualized for bacterial colony. **a.** sterile water (negative control), **b.** SL PAV B1, **c.** SL PAV B2, and **d.** *E. coli* (positive control)***.***

**b**

**a**


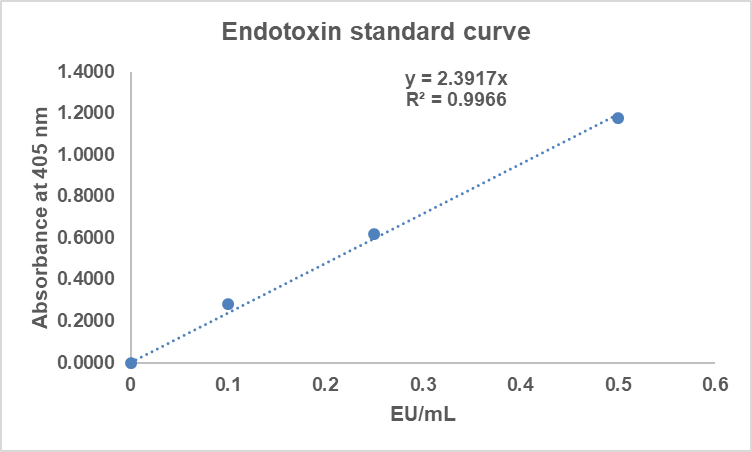


**Supplementary Fig S6.** Determination of endotoxin level in two batches of Sri Lanka PAV. Values are mean ± SD of triplicate determinations. **a.** Endotoxin standard curve of *E. coli* endotoxin. **b.** Bar diagram shows the endotoxin level in two batches of SL PAVs. No significant difference was observed between the two batches of SL PAV (p>0.05).


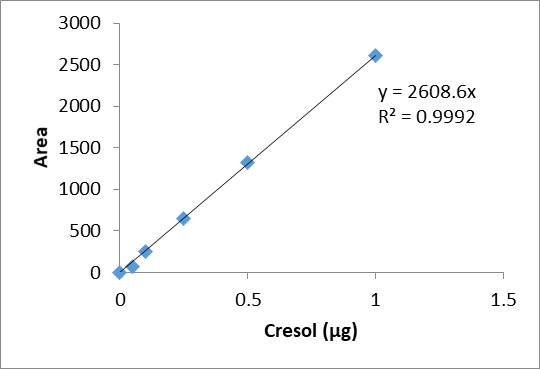


**b**


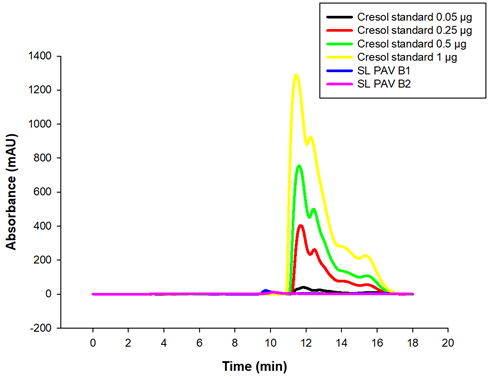


**a**


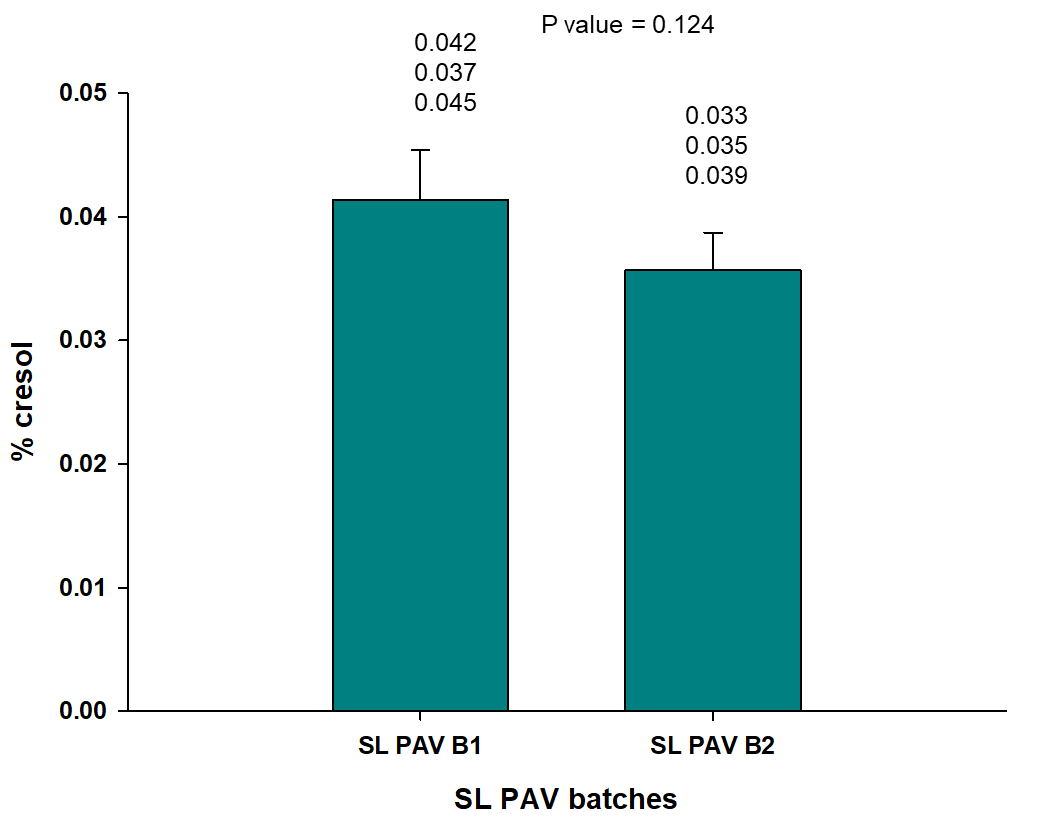


**c**

**Supplementary Fig S7.** Determination of preservative (*m*-cresol) content in different batches of PAVs. **a.** Chromatogram of the different concentration m-cresol used for prepare the standard curve from area of the peak. **b.** Standard curve prepared from the area of the m-cresol peak subjected to RP-HPLC under identical conditions. **c.** Bar diagram represents the percent m-cresol present in the two batches of SL PAVs. Values are mean ± SD of triplicate determinations. No significant difference was observed between the two batches of SL PAV (p>0.05).


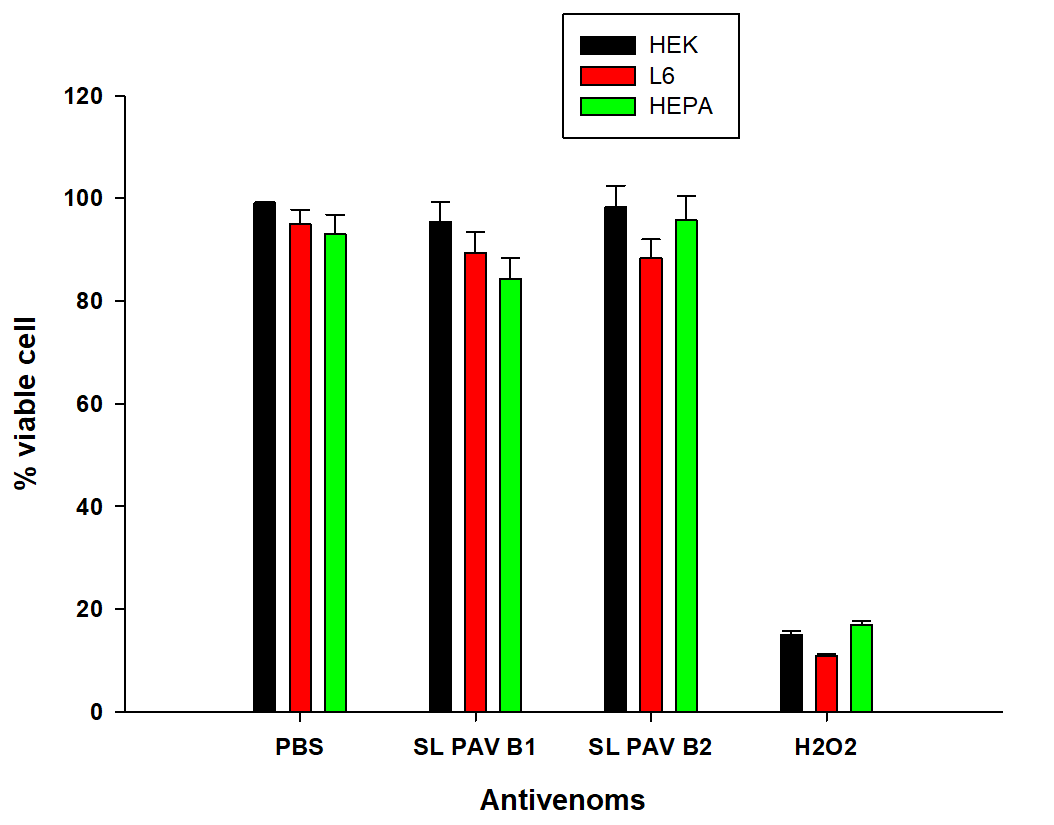


**
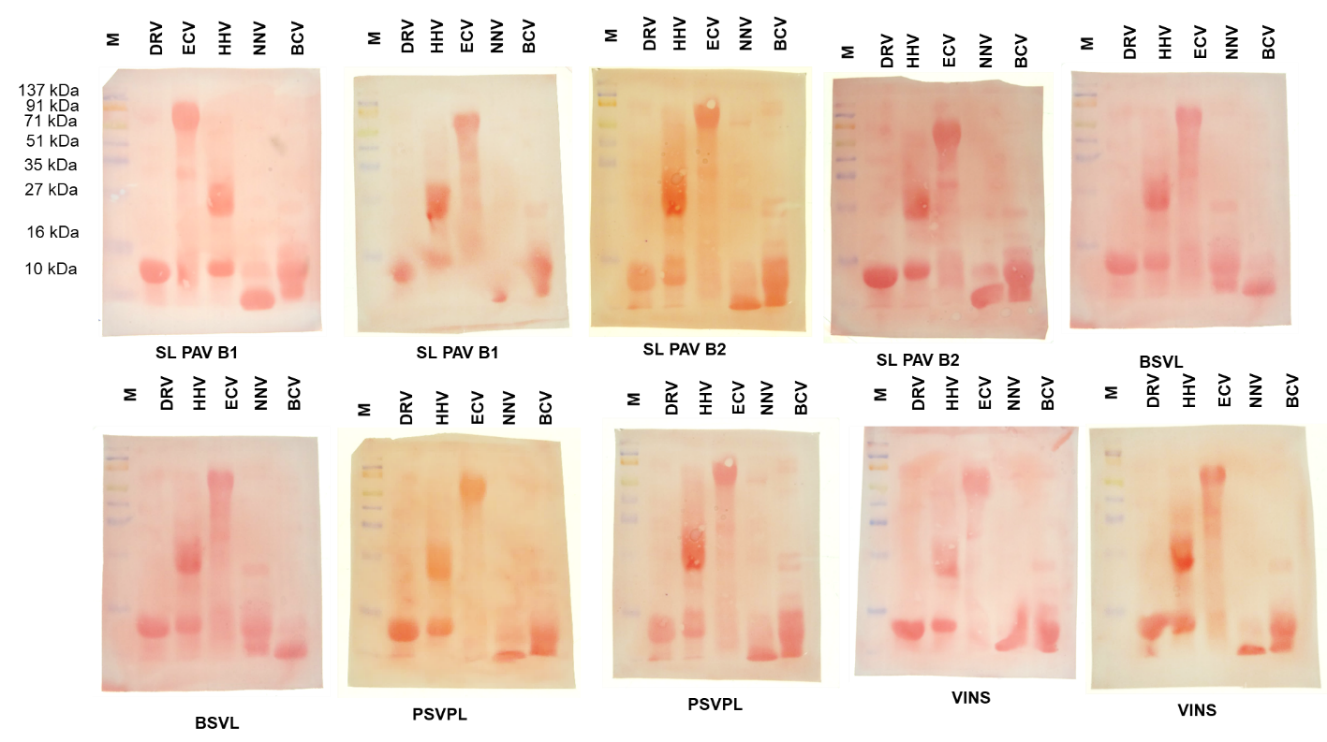
Supplementary Fig S8.** Assessment of cell cytotoxicity of Sri Lanka PAV, if any, against human embryonic kidney (HEK-293T), mouse hepatocyte (Hepa 1-6), and differentiated L6 myotubes cells. The cytotoxicity was assessed post 72 h of treatment. Phosphate buffer saline (PBS) was used as negative control and H_2_O_2_ (300 µM) was used as positive control for the assay. Values are mean ± SD of triplicate determinations. No significant difference was observed between the two batches of SL PAV (p>0.05).

**Supplementary Fig S9**. Ponceau stained image of SL snake venoms after transfer to the PVDF membrane. Same molecular marker (M) was used for all the blots.

**
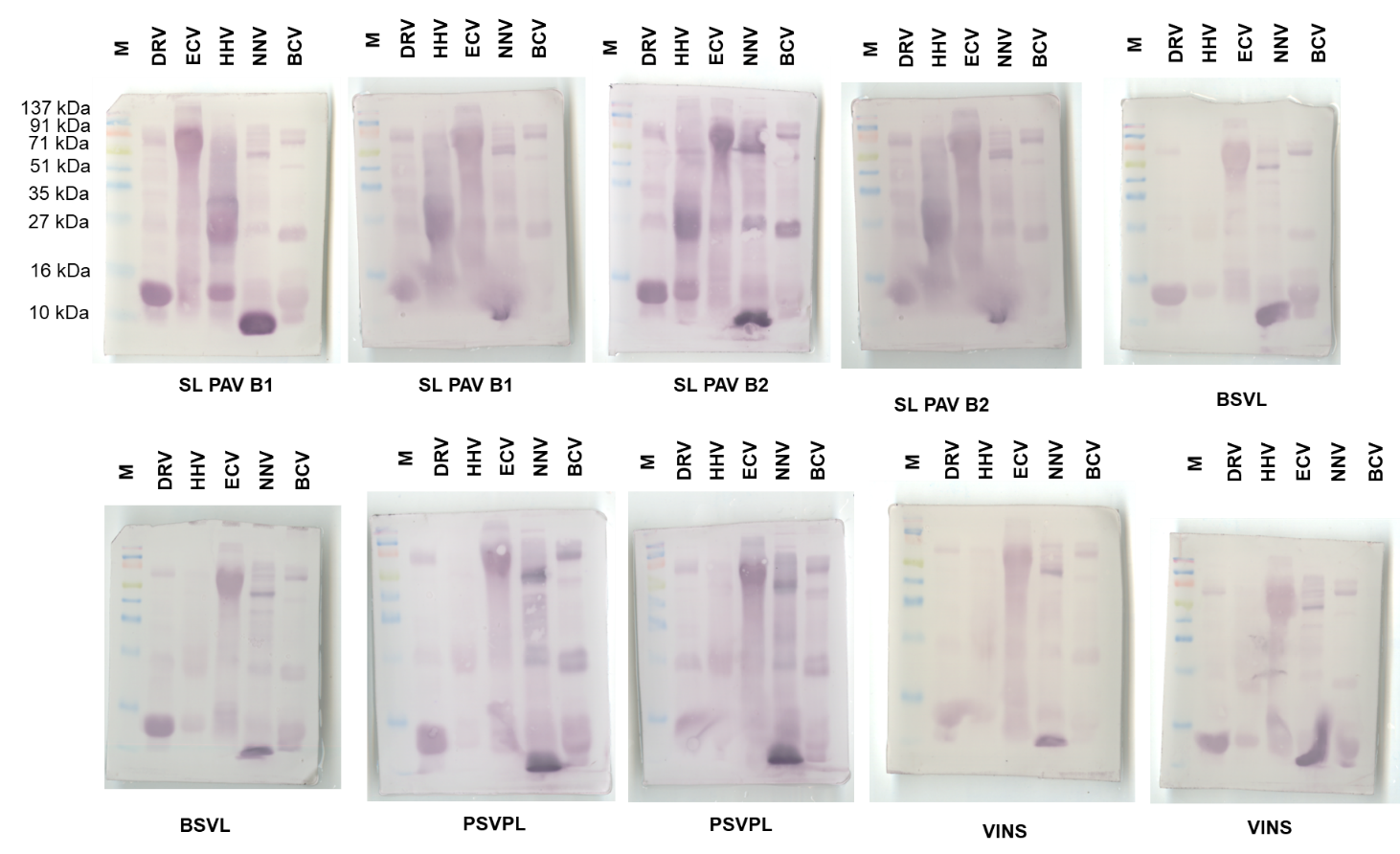
**

**Supplementary Fig S10**. Full length Western blot images of SL venoms against two batches (B1 and B2) of SL PAV and Indian PAVs (BSVL, PSVPL, and VINS) as shown in Fig. 6 Same molecular marker (M) was used for all the blots.


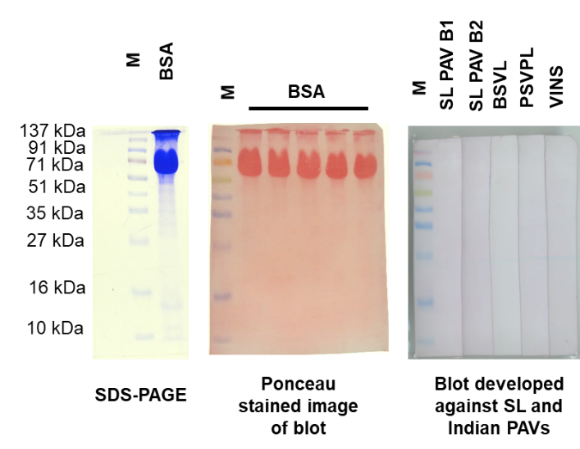


**b**

**a**


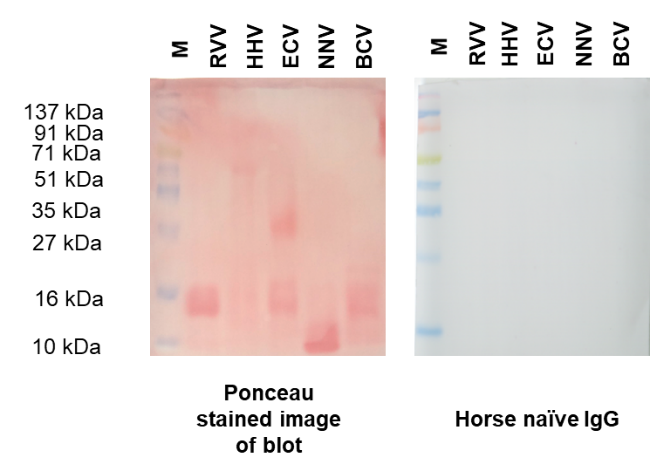


**Supplementary Fig S11. a.** Ponceau stained image of SL venom after transferring to PVDF membrane and immunoblot image of SL venoms against naïve horse IgG. **b.** SDS-PAGE image of BSA and Ponceau stained image of BSA lanes transferred to the membrane. After Ponceau staining the membranes were cut according to lanes and immunoblotting was performed against two batches of SL PAVs and Indian PAVs (negative control).


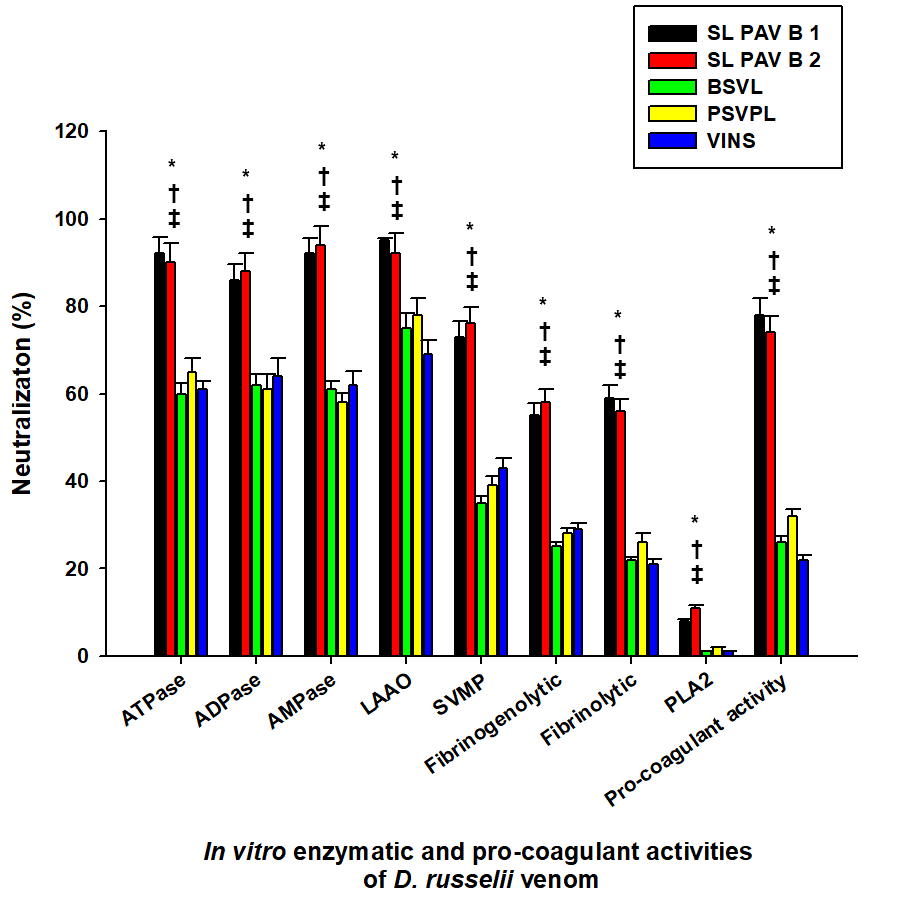


**a**


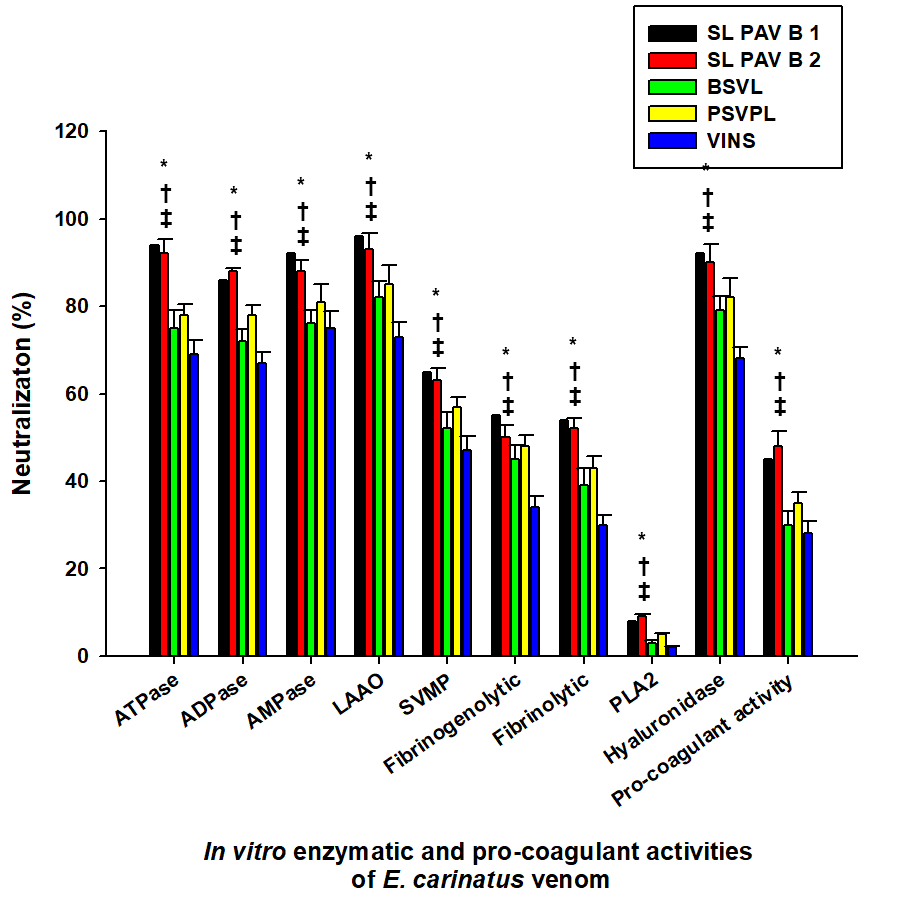


**b**


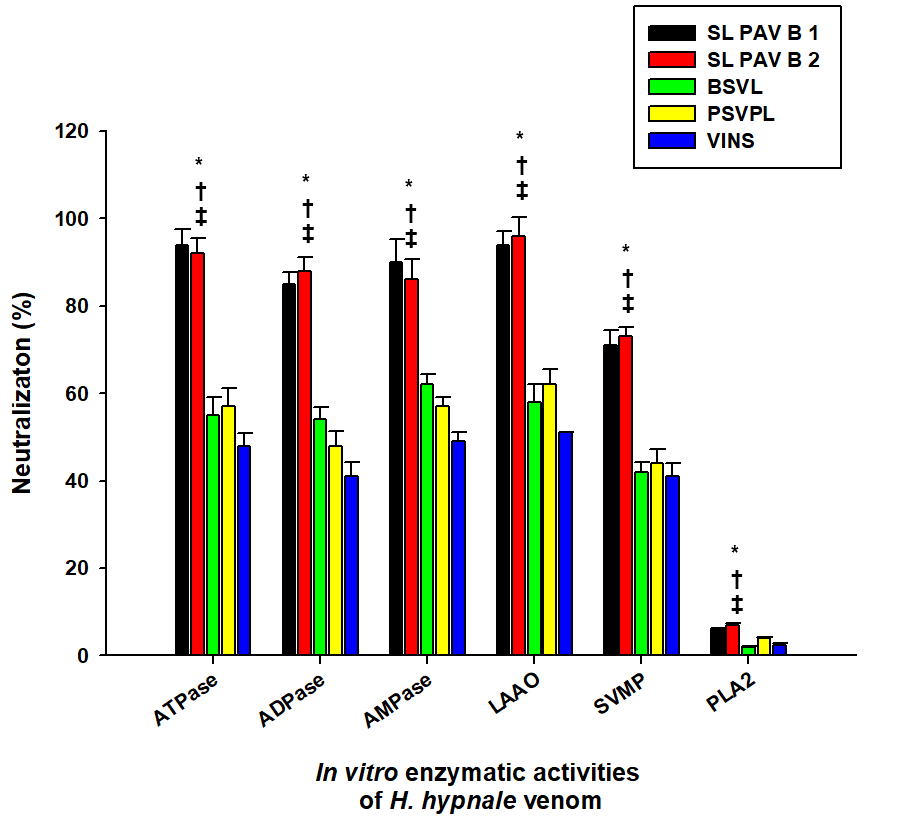


**c**


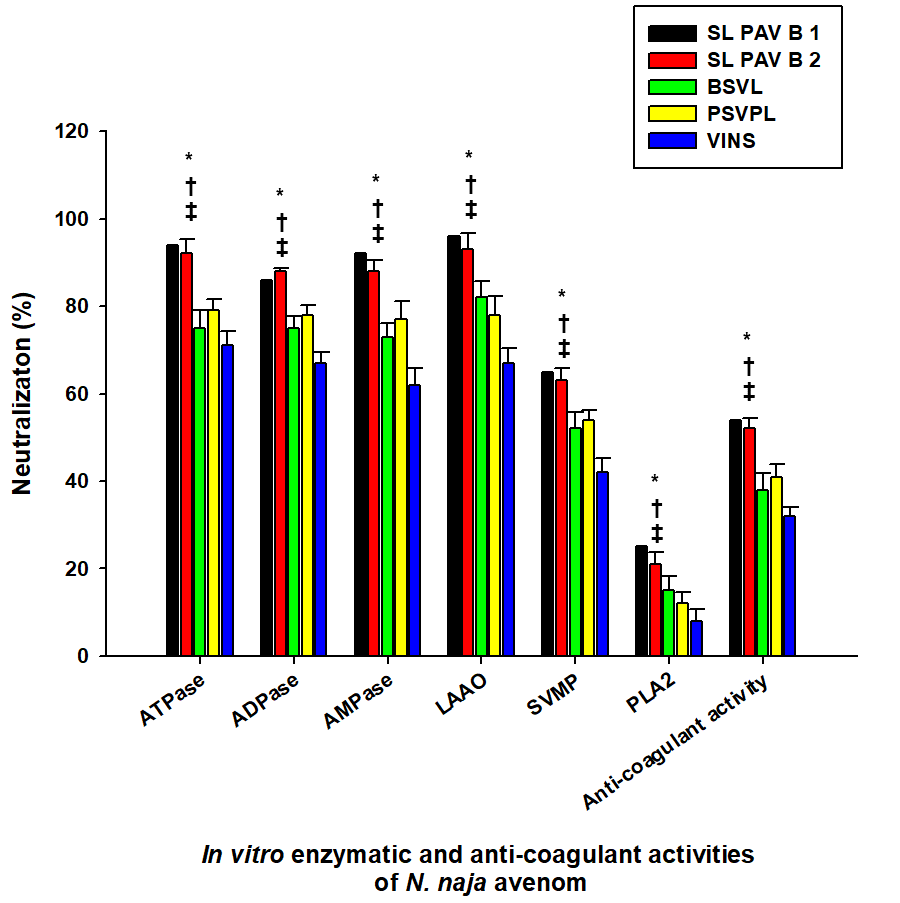


**d**


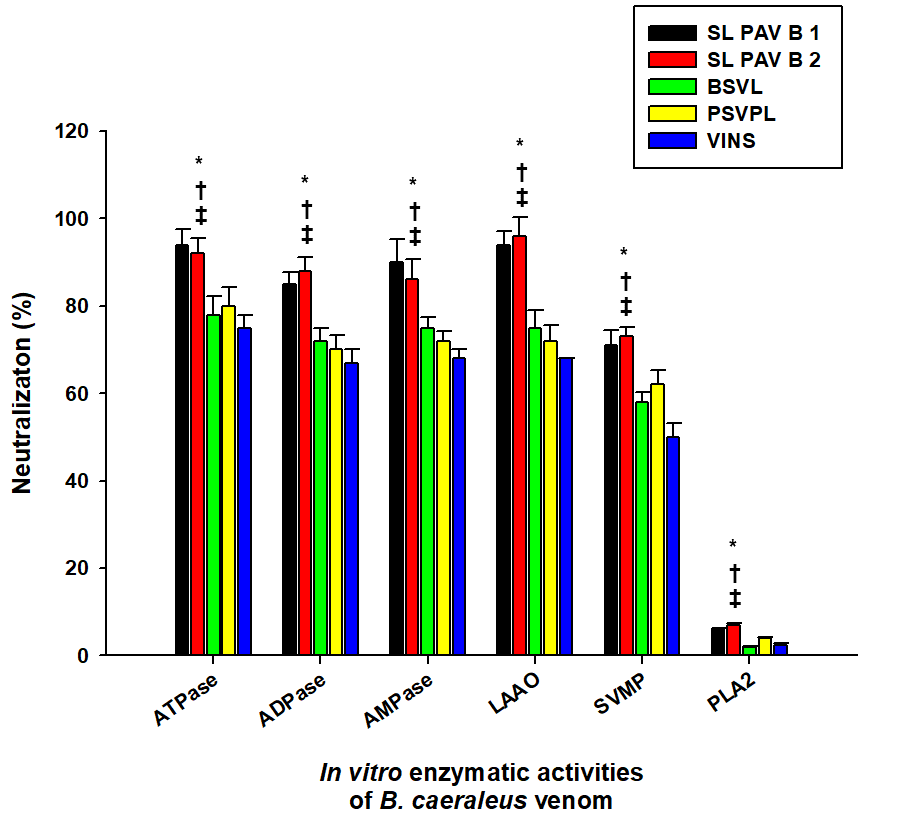


**e**

**Supplementary Fig S12.** A comparison of neutralization of some enzymatic activities and *in vitro* pharmacological properties of SL snake venoms by newly developed SL country-specific PAV and Indian PAVs. **a.** *D. russelii* venom*;* **b**. *E. carinatus* venom; **c**. *H. hypnale* venom; **d**. *N. naja* venom and **e**. *B. caeruleus* venom by two batches of SL PAVs (B1 and B2) and Indian PAVs (BSVL, PSVPL and VINS). Values are mean ± SD of triplicate determinations. *Significance of difference between SL PAV B1 and B2 with respect to BSVL; ^†^significance of difference between SL PAV B1 and B2 with respect to PSVPL; ^‡^significance of difference between SL PAV B1 and B2 with respect to VINS (p<0.05). The two batches (B1 and B2) of SL PAV did not show significant difference (p>0.05) in neutralizing the enzymatic activities and pharmacological properties of SL snake venoms.


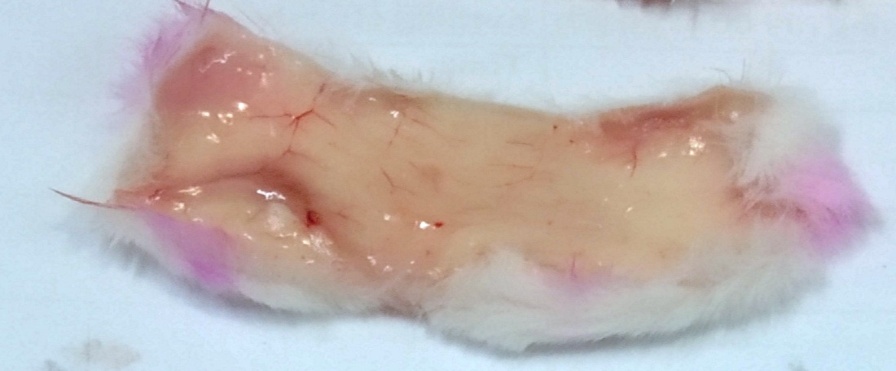
**
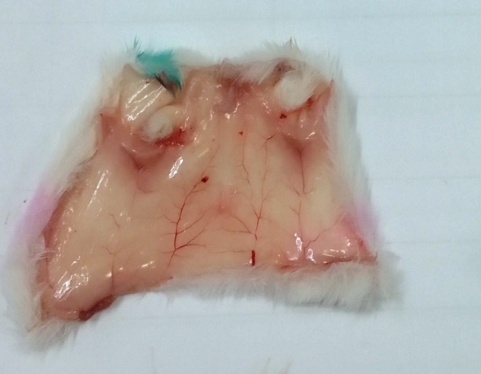
**

Photo.1 MHD Photo.2 MHD


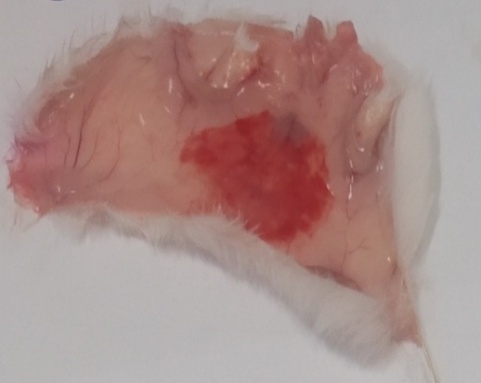

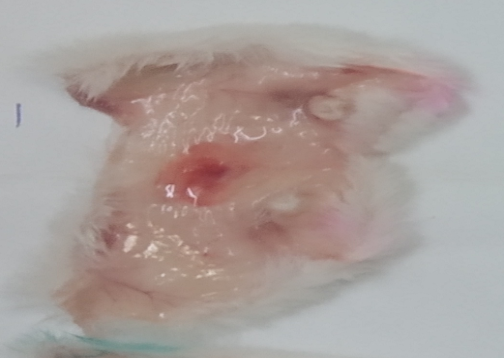


Photo.3 MHD Photo.4 MHD_50_


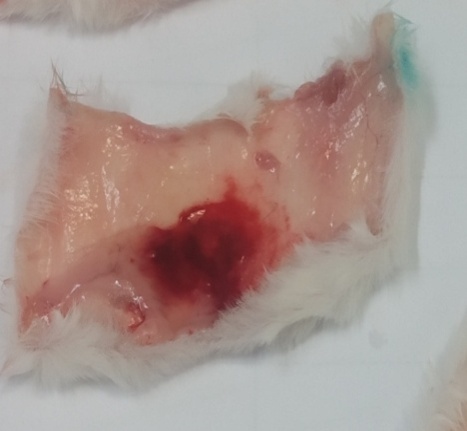

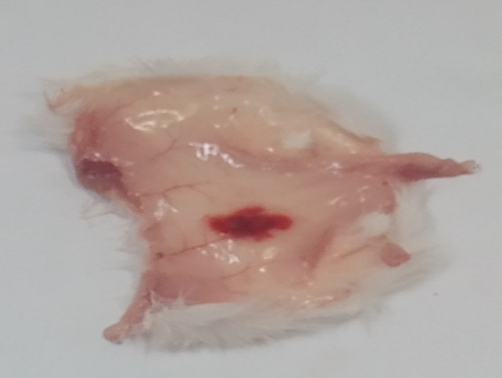


Photo.5 MHD Photo.6 MHD_50_


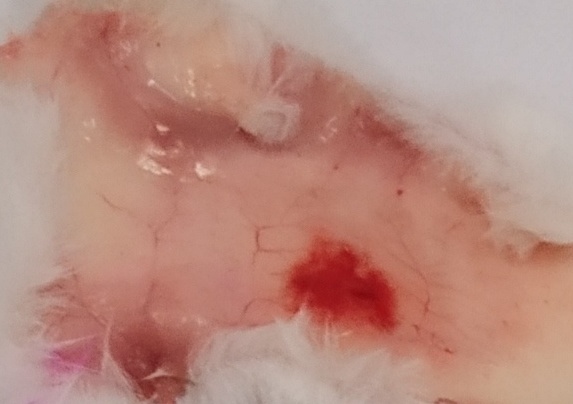

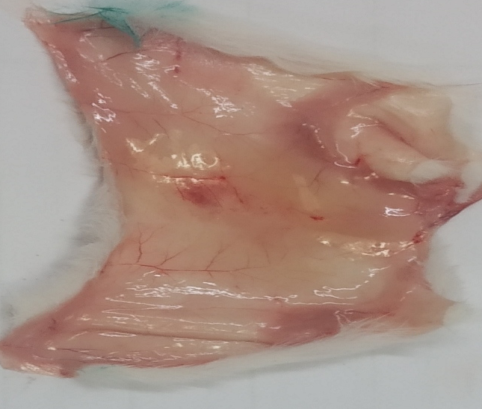


Photo.7 MHD Photo.8 MHD_50_


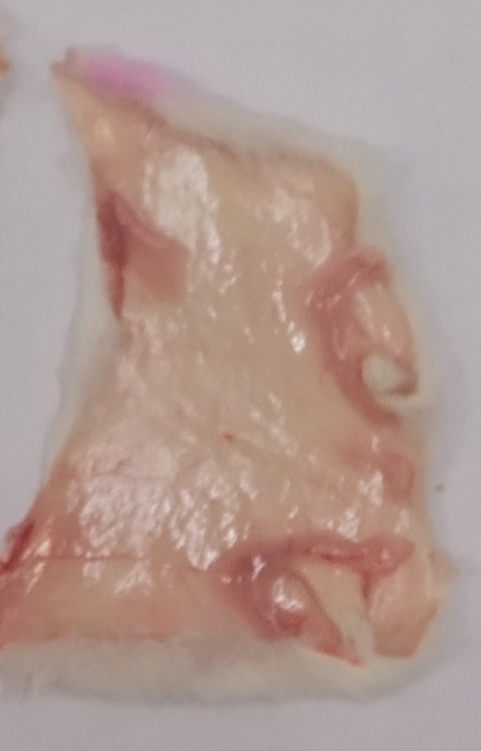

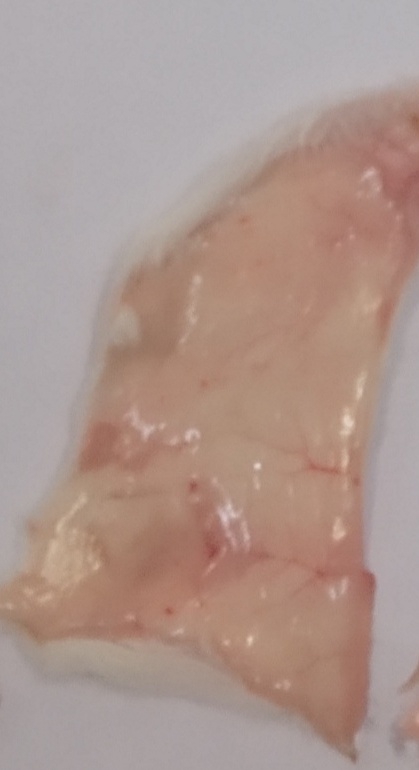


Photo.9 MND Photo.10 MND_50_


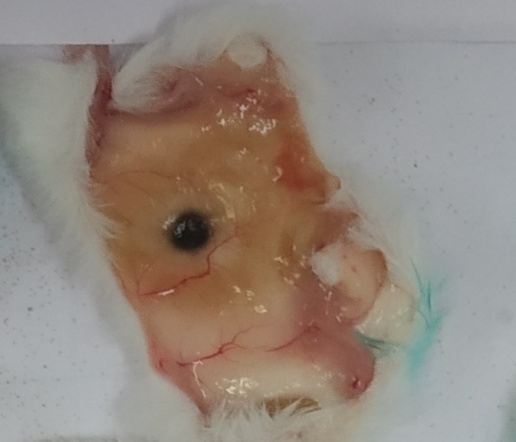

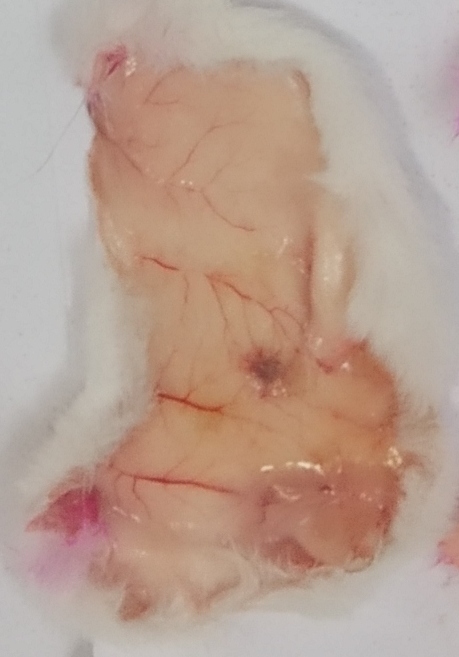


Photo.11 MND Photo.12 MND_50_


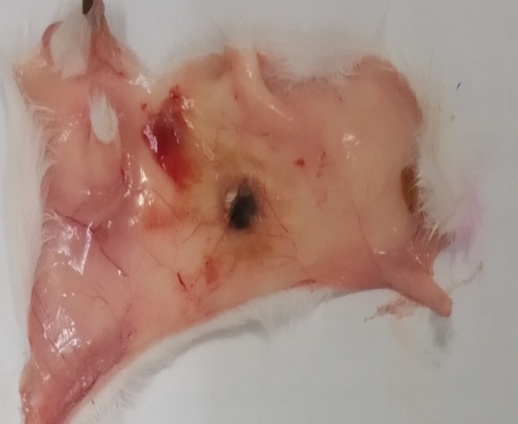

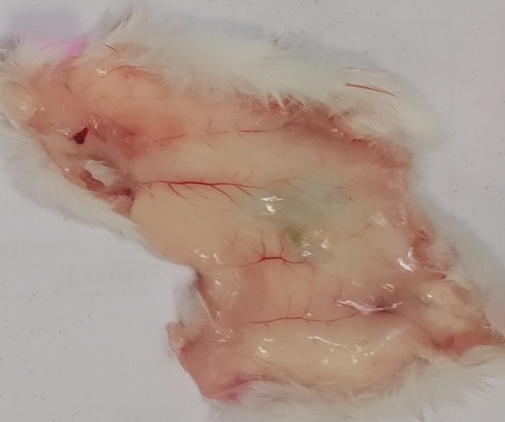


Photo.13 MND Photo.14 MND_50_


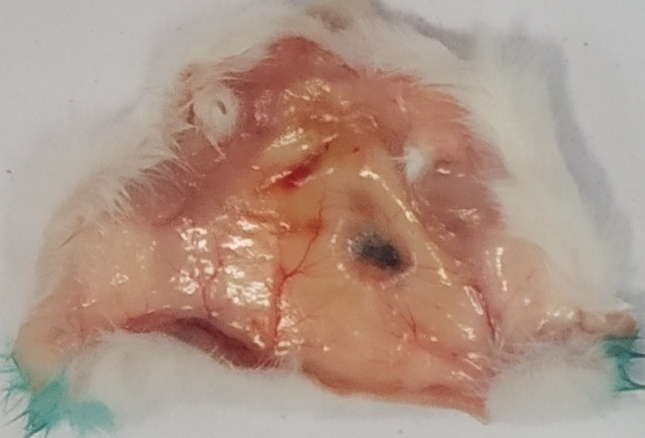

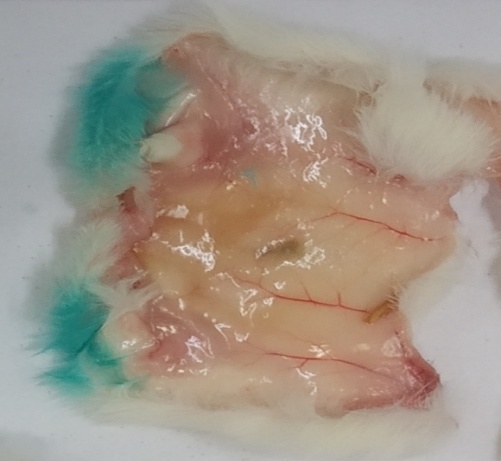


Photo.15 MND Photo.16 MND_50_

**Supplementary Fig S13.** Photograph of toxic activities of SL venoms

Photo.1 MHD photograph of *Naja naja* venom- No MHD

Photo.2 MHD photograph of *Bungarus caeruleus* venom- No MHD

Photo.3 MHD photograph of *Daboia russelii* venom- Shows haemorrhagic activity

Photo.4 MHD_50_ photograph of *Daboia russelii* venom- Shows 50% neutralization of venom-induced haemorrhage

Photo.5 MHD photograph of *Echis carinatus* venom- Shows haemorrhagic activity

Photo.6 MHD_50_ photograph of *Echis carinatus* venom- Shows 50% neutralization of venom-induced haemorrhage

Photo.7 MHD photograph of *Hypnale hypnale* venom- Shows haemorrhagic activity

Photo.8 MHD_50_ photograph of *Hypnale hypnale* venom- Shows 50% neutralization of venom-induced haemorrhage

Photo.9 MND photograph of *Naja naja* venom- No MND

Photo.10 MND photograph of *Bungarus caeruleus* venom- No MND

Photo.11 MND photograph of *Daboia russelii* venom- Shows necrotic activity

Photo.12 MND photograph of *Daboia russelii* venom- Shows 50% neutralization of venom-induced dermonecrosis

Photo.13 MND photograph of *Echis carinatus* venom- Shows necrotic activity

Photo.14 MND photograph of *Echis carinatus* venom- Shows 50% neutralization of venom-induced dermonecrosis

Photo.15 MND photograph of *Hypnale hypnale* venom- Shows necrotic activity

Photo.16 MND photograph of *Hypnale hypnale* venom-Shows 50% neutralization of venom-induced dermonecrosis

MHD: The minimum haemorrhagic dose of a venom (MHD) is defined as the amount of venom which, when injected intradermally, induces in mice a 10 mm haemorrhagic lesion after a predefined time interval, usually 2–3 hours, after injection .

MHD50: The assay measuring the efficacy of antivenom to neutralize venom-induced haemorrhage is termed the MHD-median effective dose (MHD50), and is defined as the volume of antivenom, in microlitres, or the venom/ antivenom ratio, which reduces the diameter of haemorrhagic lesions by 50% when compared with the diameter of the lesion in animals injected with the control venom/saline mixture.

MND: The minimum necrotizing dose (MND) of a venom is defined as the smallest amount of venom (in μg dry weight) which, when injected intradermally into mice shows a necrotic lesion of 5 mm diameter 3 days later.

MND50: The assay measuring the ability of antivenom to neutralize venom-induced dermonecrosis is termed the MND-median effective dose (MND50), and is defined as the volume of antivenom, in microlitres or the venom/ antivenom ratio, which reduces the diameter of necrotic lesions by 50% when compared with the diameter of the lesion in mice injected with the control venom/saline mixture.

**Reference:**

1 Patra, A., Kalita, B. & Mukherjee, A. K. Assessment of quality, safety, and pre-clinical toxicity of an equine polyvalent anti-snake venom (Pan Africa): Determination of immunological cross-reactivity of antivenom against venom samples of Elapidae and Viperidae snakes of Africa. *Toxicon* **153**, 120-127 (2018).
